# *Chlamydia*-driven ISG15 expression dampens the immune response of epithelial cells independently of ISGylation

**DOI:** 10.1128/mbio.02401-24

**Published:** 2024-09-30

**Authors:** Yongzheng Wu, Chang Liu, Chongfa Tang, Béatrice Niragire, Yaël Levy-Zauberman, Cindy Adapen, Thomas Vernay, Juliette Hugueny, Véronique Baud, Agathe Subtil

**Affiliations:** 1 Cellular Biology of Microbial Infection, Institut Pasteur, Université Paris Cité, CNRS UMR3691, Paris, France; 2 National Vaccine and Serum Institute, Beijing, China; 3 Sorbonne Université, Collège doctoral, Paris, France; 4 Service de Chirurgie gynécologique, Institut Mutualiste Montsouris, Paris, France; 5 Laboratoire NF-κB, Differentiation and Cancer, Université Paris Cité, Paris, France; National University of Singapore, Singapore, Singapore

**Keywords:** *C. trachomatis*, interferon stimulated gene 15 (ISG15), innate defense, epithelial cells, inflammation, ISGylation

## Abstract

Excessive inflammation upon *Chlamydia trachomatis* infection can cause severe damages in the female genital tract. This obligate intracellular bacterium develops mainly in epithelial cells, whose innate response contributes to the overall inflammatory response to infection. The ubiquitin-like protein interferon-stimulated gene 15 (ISG15) stimulates interferon γ (IFNγ) production and is required for bacterial clearance in several infectious contexts. Here, we describe and investigate the consequences of the increase in ISG15 expression by epithelial cells infected with *C. trachomatis*. Infection of HeLa cells and primary ecto-cervical epithelial cells resulted in a transcriptional upregulation of *ISG15* expression. This did not involve the canonical type I interferon (IFN-I) signaling pathway and depended instead on the activation of the STING/TBK1/IRF3 pathway. The absence or reduction of ISG15 synthesis led to increased production of several cytokines and chemokines, including interleukin (IL) 6 and IL8. This implicates that ISG15 normally dampens the immune response induced by *C. trachomatis* infection in epithelial cells. ISG15 exerted its control from an intracellular location, but without involving ISGylation. Finally, higher levels of inflammation and delayed bacterial clearance were observed in the genital tracts of ISG15-KO mice infected by *C. trachomatis* compared with wild-type animals; however, IFNγ production was unchanged. Altogether, our data show that ISG15 expression acts as a brake on the immune response to *C. trachomatis* infection in epithelial cells and limits bacterial burden and inflammation in mice.

**IMPORTANCE:**

Infection of epithelial cells by *Chlamydia trachomatis* elicits an innate immune response by these cells. The signaling pathways involved, and their outcomes, are still very poorly understood. In this paper, we described how *Chlamydia* infection triggered the expression of ISG15, a small molecule normally associated to type I interferon (IFN-I) signaling and control of INF-γ production. ISG15 synthesis by epithelial cells attenuated their immune response to *Chlamydia* infection. In mice, we observed that ISG15 displayed a marginal role in modulating the production of IFN-γ, a key component of the host immune response to infection, but facilitated bacterial clearance. Overall, our study strengthens the importance of ISG15 not only in the resolution of viral but also of bacterial infection and document its role of “immune brake” in the context of *Chlamydia* infection.

## INTRODUCTION

Mammalian cells possess various sensors to detect invading microorganisms and activate innate defense mechanisms, especially the secretion of inflammatory cytokines and chemokines, aimed at the elimination of the intruders. Several cellular components are involved in tuning this inflammatory response to infection. Such is the role of interferon-stimulated gene 15 (ISG15), a protein of 15 kDa with two ubiquitin-like domains. Similar to ubiquitin, it can be conjugated to a lysine residue on a target protein through a series of enzymatic reactions involving E1/E2/E3 ligases. ISG15 conjugation (ISGylation) is rendered possible by the LRLRGG motif exposed after the removal of eight amino acids at the C-terminus of the protein (1). ISG15 also displays biological activity as a free molecule from two locations; it can be secreted and exert its activity through binding to a specific membrane receptor or it can act as a free cytoplasmic protein by unknown mechanism(s) (2–4). ISG15 and the members of the enzymatic cascade that lead to ISGylation are strongly induced by type I interferon (IFN-I, e.g., IFNa and IFNb). ISG15 was initially studied for its anti-viral property in mice (5); however, against expectations, inherited ISG15-deficient patients do not show increased susceptibility to viral infection. Conversely, weakly virulent mycobacteria, such as *Mycobacterium bovis* Bacille Calmette-Guérin (BCG), which only trigger a mild inflammatory response in normal individuals, induce a life-threatening infection in patients lacking ISG15 (6). It was shown that lack of ISG15-dependent induction of IFNγ was responsible for the severe consequences of ISG15 deficiency in humans (6). Later work showed that, unlike in mice, ISG15 deficiency increased viral resistance in humans, and that ISG15 acted in a free form in antimycobacterial immunity (7). Additionally, it was reported that ISG15 expression was induced independently of IFN-I upon infection of epithelial cells by *Listeria monocytogenes*, a Gram-positive bacterium that developed in the cytoplasm (8). Similarly to *Mycobacterium'*s case, ISG15 expression in human cells restricted *Listeria* development, supporting the idea that this ubiquitin-like molecule was exerting anti-bacterial activity(ies).

*Chlamydia trachomatis* is an interesting infectious bacterium to further explore the role of ISG15 in the innate host response to bacterial infection, since it is a Gram-negative bacterium that develops in a different cell type than *Mycobacterium*, and in a vacuole, unlike *Listeria. C. trachomatis* is the leading cause of bacterial sexually transmitted infections (STIs) in the world and of preventable blindness of bacterial origin, and constitute one major problem for public health worldwide (9). This obligate intracellular bacterium grows exclusively inside a vacuolar compartment, the inclusion, within the epithelial cells of genital or conjunctival mucosae. Genital *C. trachomatis* infections are very common, with a worldwide estimate of more than 100 million new cases every year (10). The prevalence is difficult to record because most cases are asymptomatic and go undetected. Chronic and/or repeated infection can eventually cause severe and irreversible consequences, particularly in women, such as pelvic inflammatory disease and scar formation in the fallopian tubes leading to tubal infertility. Although tissue damages may be due to inadequate levels of immune response of the host, the conditions that favor these deleterious consequences are still poorly understood.

The discovery that the innate immune response was sufficient for eradicating *C. trachomatis* from the genital tract of mice highlighted its importance for clearing *Chlamydia* infection (11). Similar to other microorganisms, *C. trachomatis* have acquired several mechanisms to subvert host defense (12). For instance, the bacteria express a protease *Chlamydia* protease-like activity factor (CPAF) that is capable of blocking the secretion of neutrophil extracellular traps by neutrophils (13), of degrading host antimicrobial peptides (14), and, possibly indirectly, of inhibiting NF-κB p65 translocation to the nucleus (15). The bacteria also secrete a deubiquitinase that inhibits ubiquitination primed by IFNγ in human epithelial cells, thereby evading subsequent elimination by the host (16). The translocated early phosphoprotein (TepP), an effector of *C. trachomatis* which is secreted at early stage of infection, can also manipulate the host signals associated with innate immunity of host cells (17, 18).

Low- and high-risk human papilloma virus (HPV) infections elicit differential ISG15 induction in the cervical mucosae of infected human patients, indicating that ISG15 may contribute to the innate response to infection in the female genital tract (FGT) (19). Furthermore, given the key role of IFNγ in resolving *C. trachomatis* infection (20–22) and the fact that ISG15 is described as a potent IFNγ-inducing cytokine playing an essential role in antimycobacterial immunity (6), we anticipated that ISG15 might be an important player for efficient clearing of *C. trachomatis* infection. Indeed, we observed a strong induction of ISG15 expression by epithelial cells upon infection by *C. trachomatis*. We uncovered the signaling cascade that led to an increase in *ISG15* transcription and studied its outcome on infection.

## MATERIALS AND METHODS

### Cells

The human cervical epithelial HeLa cell line was from ATCC (CCL-2). The cells were grown and propagated in Gibco Dulbecco’s Modified Eagle Medium (DMEM) with GlutaMAX (Thermo Fisher Scientific) and 10% of fetal calf serum (FCS) at 37°C with 5% of CO_2_. Primary ecto-cervical epithelial cells were isolated as described previously (23) and cultured in Gibco keratinocyte serum-free medium (K-SFM) supplemented with recombinant human EGF and bovine pituitary extract (Thermo Fisher Scientific, #17005075). The cells were propagated in 75 cm^2^ flasks (Falcon) and seeded in culture dish (0.1 × 10^6^ cell/well for 24-well plates or 0.2 × 10^6^ cell/well for 12-well plates) 24 h before infection or treatment.

ISG15-KO HeLa cells were obtained following a standard procedure (24). Briefly, 0.5 µg of pSpCas9(BB)-2A-Puro (PX459) V2.0 plasmid (Addgene #62988) containing guide RNA (gRNA) duplex sequence of human ISG15 (F: 5’-ggcgtgcacgccgatcttct; R: 5’- agaagatcggcgtgcacgcc) inserted with BbsI enzyme, was transfected into HeLa cells (0.15 × 10^6^ cells/well, 24-well plate) seeded overnight before transfection using 1 µL of jetPRIM (Polyplus). After 4 h, the medium was replaced by fresh medium containing puromycin (2 µg/mL), and cells were incubated for 24 h. The surviving cells were then detached, counted, and serially diluted to collect individual clones in 96-well plates. The absence of ISG15 expression was verified on individual clones by Western blot.

### Bacteria

*C. trachomatis* serovar LGV-L2 (434/Bu), LGV-L2^IncD^GFP and LGV-L2^IncD^mCherry bacteria expressing constitutively GFP and mCherry, respectively (25, 26), were purified on a density gradient as previously described (27). In brief, 80% confluent HeLa cells were infected with LGV-L2 (MOI = 1) in the presence of cycloheximide (1 µg/mL). After 48 h, the cells were homogenized mechanically with sterile glass beads, followed by sonication to release bacteria. Infectious bacteria were purified by density-gradient centrifugation using Gastrografin (Bayer, Germany), resuspended in sucrose–phosphate–glutamic acid (SPG) buffer (10 mM sodium phosphate [8 mM Na_2_HPO_4_-2 mM NaH_2_PO_4_], 220 mM sucrose, 0.50 mM l-glutamic acid), aliquoted and stocked at −80°C.

### siRNA treatment

siRNA smartpools were purchased from Dharmacon (Lafayette, USA) except for siRNAs for ISG15, which were individually purchased from Eurogentec (Seraing, Belgium). One and a half microliter siRNA (10 µM) was mixed with 0.5 µL Lipofectamine iMax (Invitrogen) in 50 µL of Opti-MEM media (Gibco) for 10 min at room temperature according to the manufacturer’s recommendation. The mixture was added into a culture dish before adding cells (0.1 × 10^6^ cells/well for 24-well plate) suspended in 0.45 mL DMEM/FCS medium (final concentration of siRNA was 30 nM), mixed and incubated for 48 h before treatment or infection. The siISG15 oligos were mixed (10 µM each) #1 5′-uccuggugaggaauaacaa dTdT-3′; #2 5′-gcaccguguucaugaaucu dTdT-3′) (28).

### Cell infection

Cells, seeded the previous day in culture dish (0.1 × 10^6^ cell/well for 24-well plate or 0.2 × 10^6^ cell/well for 12-well plate), were rinsed twice using pre-warmed media before adding LGV-L2 in the culture media. After 24 to 42 h later, the culture medium was collected, spinned for 15 min at 1,500×*g* to remove cell debris, frozen at −20°C and used for later cytokine assay by enzyme-linked immunosorbent assay (ELISA). The cells were lysed either in urea buffer (8 M urea, 30 mM Tris, 150 mM NaCl, 1% v/v sodium dodecyl-sulfate pH 8.0) for protein extraction or in RLT buffer (Qiagen) for RNA extraction. In certain experiments, the cells were pre-treated with pharmacological inhibitors for 30 min or transfected with siRNA for 48 h before bacterial infection. Wortmannin was from Selleckchem (#S2758).

### Progeny assay

Cells (0.1 × 10^6^ cells/well) plated the day before in 24-well plates were infected with LGV-L2^IncD^mCherry bacteria expressing constitutively mCherry at a MOI = 0.15. After 48 hpi, the cells were detached, lysed using glass beads, and the supernatant was used to infect HeLa cells plated the day before (0.1 × 10^6^ cells/well in a 24-well plate), in serial dilutions. After 24 h, the cells with an infection <30% (checked by microscopy) were detached using 0.5 mM EDTA in PBS and fixed in 4% (w/v) paraformaldehyde (PFA) 4% (w/v) sucrose in PBS before analysis by CytoFLEX flow cytometer (Beckman Coulter). The data were analyzed using FlowJo (version 10.0.7) to determine the bacterial titer.

### Bacterial adhesion and entry studies

*C. trachomatis* binding to or entry into epithelial cells was performed as described previously (25). For binding experiments, pre-chilled cells (30 min at 4°C) grown on coverslips were incubated with LGV-L2^IncD^GFP (MOI = 30) for 4 h at 4°C. Bacteria were gently sonicated before infection to disrupt bacterial aggregates. The cells were washed thrice with chilled DMEM medium and fixed with 4% (w/v) paraformaldehyde (PFA) and 4% (w/v) sucrose for 20 min at room temperature permeabilized by 0.05% saponin/PBS/0.1% BSA solution. DNA was stained by Hoechst (0.5 µg/mL) before manually counting GFP bacteria per cell in several fields taken with a Deltavision microscope (GE, UK). For bacterial entry study, pre-chilled cells seeded on coverslip were incubated with LGV-L2^IncD^GFP (MOI = 10) for 45 min at 4°C. The cells were then washed with pre-warmed medium thrice and incubated at 37°C for the indicated time before fixation as above. Extracellular bacteria were stained with a mouse anti-MOMP-LPS antibody (Argene #11–114) followed with Cy5-conjugated anti-mouse secondary antibody (Amersham Biosciences). The bacterial entry was presented as ratio between intracellular bacteria (GFP only dots) and total bacteria (all GFP positive dots including GFP/Cy5 dots).

### Immunofluorescence

Cells (0.1 × 10^6^ cells/well) seeded on coverslips in a 24-well culture dish were infected with LGV-L2 strain with MOI = 1 for 40 h or treated with IFNα (Biogen Idec, USA) for 24 h. The cells were then fixed in PFA as described above. After fixation, the cells were washed in PBS and incubated with 50 mM NH_4_Cl to quench the residual PFA, followed with 10 min in 0.05% (w/v) saponin, 0.1% (w/v) BSA for cell permeabilization. Intracellular bacteria and ISG15 were stained with a mouse anti-MOMP-LPS (Argene # 11–114) antibody and a rabbit anti-human ISG15 antibody (a kind gift from E. C. Borden, Cleveland Clinic, Cleveland, OH, USA) followed with Alexa488-conjugated anti-mouse and Cy5-conjugated anti-rabbit secondary antibody. The dilutions were made in PBS containing 1% of BSA and 0.05% saponin. DNA was stained using 0.5 µg/mL of Hoechst 33342 (Thermo Fisher Scientific) added in the secondary antibody solution. Coverslips were mounted on slides in a Mowiol solution. Images were acquired on an Axio observer Z1 microscope equipped with an ApoTome module (Zeiss, Germany) and a 63× Apochromat lens. Images were taken with an ORCA-flash4.0 LT camera (Hamamatsu, Japan) using the software Zen. The images of ISG15-KO cells complemented with *ISG15* gene using a sleeping-beauty system were directly observed under an Axio Observer microscope equipped with a filter to record GFP fluorescence (Zeiss, Germany). TNFa used in Fig. S5 was from R&D system (#210-TA-005/CF).

### Complementation of ISG15-KO cells by constitutively over-expressing ISG15

ISG15 sequence with full-length or depletion of six nucleotides encoding two glycine residues at the C-terminus of the protein was cloned into pSBbi-GP plasmid (addgene, #60511) by Gibson assembly and verified by sequencing. In this plasmid, the same constitutive promoter controls expression of GFP and ISG15. The plasmids were transfected into ISG15-KO cells using jetPRIME transfection reagent (Polyplus), and the culture medium was replaced 4 h later. At 24 h post-transfection, the transfected cells were selected by puromycin (1 µg/mL). The medium containing puromycin was replaced every 2 days to remove dead cells. Selection efficiency was evaluated under the microscope by monitoring GFP expression. At 7 to 10 days after puromycin selection, almost all cells were GFP-positive and cell pools were frozen or kept in culture for the experiments.

### RT-PCR and quantitative PCR

Total RNAs were isolated from infected or treated cells using RNeasy Mini Kit (Qiagen), and reverse transcription (RT) was performed using the M-MLV Reverse Transcriptase (Promega). The genomic DNA of mouse tissue was extracted using DNeasy Blood & Tissue Kit (Qiagen). Briefly, the frozen upper FGT was homogenized in pre-chilled Lysing Matrix D 2 mL tube (MP Biomedicals) containing 400 µL PBS using Precellys 24 tissue homogenizer (Bertin, Germany). After pelleting the tissue debris (14,000×*g*, 2 min), an aliquot of the supernatant (200 µL) was used to extract genomic DNA. The qPCR was conducted on the complementary DNA (cDNA) or genomic DNA with LightCycler 480 system (Roche) using SYBR Green Master I (Roche). For bacterial load detection, serial dilutions of known quantities of 16S DNA of *C. trachomatis* and mouse β-actin DNA were used as standardized templates for qPCR. Data were analyzed using the 2^-ΔΔCt^ method, and results were presented as Log2 fold changes compared with uninfected control (29), or as ratio of *Chlamydia* 16S (pg) to host DNA (ng) for the bacterial load. The primers used for qPCR are described in Table S1.

### Western blot, dot blot, and ELISA assays

Equal volumes of cell lysates were subjected to SDS-PAGE, transferred to polyvinylidene difluoride (PVDF) membranes, and immunoblotted with the proper primary antibodies diluted in 1× PBS containing 5% milk and 0.01% Tween-20. The primary antibodies used were rabbit anti-human ISG15, rabbit anti-heat shock protein 60 of *C. trachomatis* (in house preparation), rabbit anti-Akt (Cell Signaling, #4691) and anti-p-AKT antibodies (Cell Signaling, #4060), and mouse anti-human β-actin (Sigma, #A5441). Immunoblots were analyzed using horseradish peroxidase secondary antibodies, and chemiluminescence was analyzed on an Amersham ImageQuant 800 imaging system (Cytiva, USA).

To assess ISG15 secretion by HeLa cells, 1 mm of cell-free culture medium collected from 12-well plate was loaded by several times to a PVDF membrane using the Convertible Filtration Manifold System (Gibco-BRL, # 11055) followed with immunobloting as described above. Recombinant human ISG15 (R&D systems, #UL-601–500) resuspended in culture medium served as a positive control in this experiment.

The human XL cytokine luminex performance assay 44-plex fixed panel (R&D systems, #LKTM014) was used to measure cytokine secretion in the culture medium. The culture supernatant was spined and diluted five times in fresh medium, a 50-µL aliquot was used for ELISA assay following the manufacturer instructions. To determine cytokine/chemokine concentrations in the genital tracts of mice, the homogenate of mouse tissue was prepared as described above. Non-diluted supernatant of tissue homogenate (50 µL) was applied to Bio-plex pro mouse cytokine and chemokine assay kit (Bio-Rad, #M60-009RDPD).

### Single cell cytokine detection by flow cytometry

Cells (0.2 × 10^6^ cells/well) plated the day before in 12-well plates were infected with LGV-L2^IncD^GFP bacteria (0.1 µL) expressing constitutively GFP. At 24 hpi, the cells were incubated with 5 µg/mL brefeldin A (Biolegend) for 6 h to block cytokine secretion. The cells were then detached using 0.5 mM EDTA in PBS and fixed in 4% (w/v) paraformaldehyde (PFA) 4% (w/v) sucrose in PBS, before permeabilization with 0.3% (v/v) Triton X-100 in PBS for 10 min and block in 1% BSA in PBS for 1 h. The cells were then incubated 0.1% BSA in PBS with or without 1/40 dilution of PE/Cyanine7-conjugated anti-human IL6 (Biolegend, #501119) or 1/80 dilution of PE-conjugated anti-human IL8 (Biolegend, #511408) for 1 h. After washes, the cells were resuspended in PBS, and single-cell fluorescence was measured on a CytoFLEX flow cytometer (Beckman Coulter). Cells not stained or stained with single color were used for fluorescence compensation. The data were analyzed using FlowJo (version 10.0.7), and the data from the IL6^+^/GFP^+^ or IL8^+^/GFP^+^ populations were exported with scale values for further analysis by Prism9 (GraphPad).

### Mice and infection

Female c57BL/6 J mice were purchased from Charles River Laboratories (France). ISG15-KO mice, also in the c57BL/6J background, were kindly provided by Dr Klaus-Peter Knobeloch (Leibniz-Institut für Molekulare Pharmakologie (FMP), Germany). The animals were 6–7 weeks old. All animals were given 2.5 mg of medroxyprogesterone (Depo-provera, Pfizer) s.c. 7 days before infection to synchronize the menstrual cycle. The experiments were performed in accordance with the French national and European laws regarding the protection of animals used for experimental and other scientific purposes. The study was approved by the ethics committee of Institut Pasteur (approval N° 2014–0054). The animals were anesthetized by intraperitoneal injection of ketamine/xylazine suspended in PBS. *C. trachomatis* (10^6^ IFU/mouse) suspended in 5 µL of SPG was introduced directly to the uterine horn through non-invasive trans-cervical injection using NSET device (ParaTechs Cat # 60010). This procedure was previously shown to induce chronic infection in the upper FGT of mice (30). The same volume of SPG was injected in the upper FGT of control animals. At the indicated times post-infection, the animals were sacrificed by cervical dislocation. The upper genital tracts (uterine horn, fallopian tubes, and ovaries) were excised and frozen immediately into liquid nitrogen. For morphological changes, 4 weeks post-infection, the excised FGTs were rinsed in PBS and imaged using a Nikon camera (D700 with the Micro-Nikkor 105 mm objective). The hydrosalpinx score of the uterine horn was quantified as described (31).

### Statistical analysis

Data were analyzed using Prism9 (GraphPad). Non-paired *t*-tests, paired *t*-tests, and one-way analysis of variance (ANOVA) with *post hoc* Tukey tests were used for two-group and multiple group comparisons, respectively. The correlation between cytokines and bacterial loads was determined with two-tailed Pearson test.

## RESULTS

### *Chlamydia* infection enhances ISG15 expression in epithelial cells

**Fig 1:**
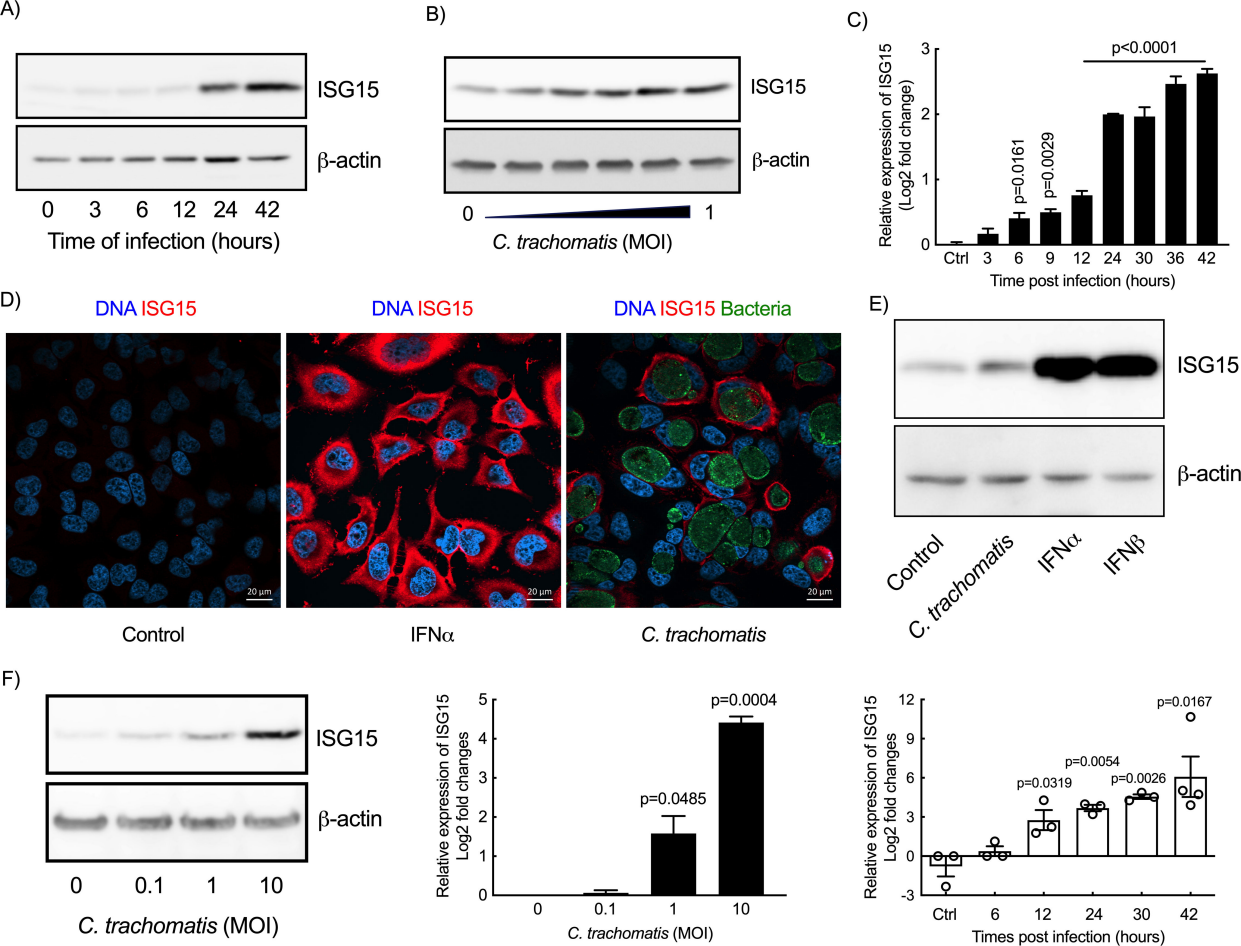
*C. trachomatis* infection induces ISG15 synthesis in epithelial cells. (A and C) Time course of infection of HeLa cells with *C. trachomatis* (A and C, MOI = 1) or with increasing MOI (B, 42 hpi). After infection, ISG15 transcript (**C**) and protein (**A-B**) levels were examined by RT-qPCR and Western blot, respectively. (**D**) HeLa cells were incubated with IFNα (10 ng/mL) or *C. trachomatis* (MOI = 1) for 30 h followed by immunostaining. (**E**) HeLa cells were incubated with *C. trachomatis* (MOI = 1) for 42 h or IFN-I (IFNα/β, 10 ng/mL for each) for 24 h before determining the expression of ISG15. (**F**) Primary epithelial cells from patients were incubated with *C. trachomatis* at the indicated MOI. ISG15 expression was examined 42 h post-infection (left and middle panels) or at the indicated times post-infection (right panel). The Western blots and immunofluorescence data are representative of at least two independent experiments. All other data represent three independent experiments. One-way ANOVA test (time course) and unpaired *t*-test (dose course in primary cells) were performed, and the p-value of the comparison with uninfected control is shown.

ISG15 expression during *C. trachomatis* infection was first examined in HeLa cells, a cancer cell line derived from the cervical epithelium. Infection of HeLa cells with *C. trachomatis* serovar L2 (LGV-L2) led to an increase in ISG15 levels in a time- and dose-dependent manner (Fig. 1A and B), which correlated with an increase in *ISG15* transcripts 6 h post-infection (hpi) and onwards (Fig. 1C). The increase in ISG15 levels in infected cells was also detected by immunofluorescence (Fig. 1D). Comparison with IFNα and IFNb, well-established inducers of *ISG15* transcription, showed that, although robust, ISG15 levels upon infection were lower than upon exposure to 10 ng/mL IFNα/b (Fig. 1E). An increase in *ISG15* transcription and higher ISG15 protein levels were also observed in primary cervical epithelial cells isolated from patient explants (Fig. 1F). We concluded from these observations that ISG15 synthesis by epithelial cell is increased upon infection by *C. trachomatis*.

### ISG15 dampens *C. trachomatis*-induced immune response

**Fig 2:**
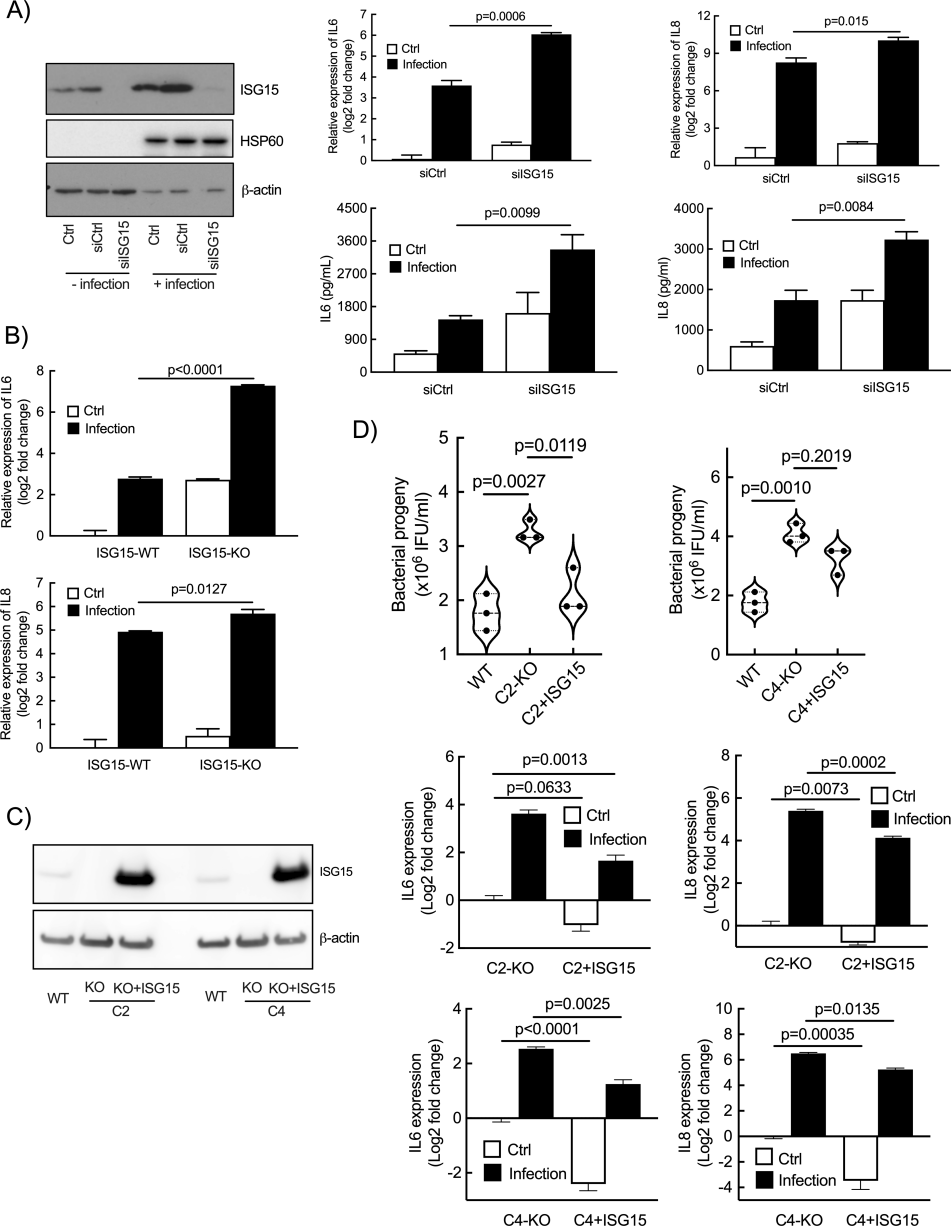
ISG15 exerts a negative control on *IL6* and *IL8* expression. (**A**) Cells were transfected with siRNA targeting human *ISG15* or non-relevant oligonucleotides for 48 h before infection with *C. trachomatis* (MOI = 1). Cells were collected at 42 hpi. ISG15 levels were determined by Western blot (left panel). HSP60, a bacterial protein, control for bacterial load, and actin serves as a loading control. The *IL6* and *IL8* transcript (top histograms) and protein (bottom histograms) levels were determined by RT-qPCR and ELISA, respectively. (**B**) MEFs lacking ISG15 or not were infected with *C. trachomatis* (MOI = 1), and the amount of *IL6* and mouse *IL8* (KC) transcripts were determined at 42 hpi by RT-qPCR. (**C**) *ISG15*-deficient clones (C2 and C4) were complemented with ISG15 (left panel) and infected with *C. trachomatis* (MOI = 1). Transcript levels for *IL6* and *IL8* were measured at 42 hpi (middle and right panels). (**D**) Quantification of infectious bacteria collected in the indicated cellular backgrounds at 48 hpi. Each dot in panel D represents an individual experiment. The rest of the data are representative of at least three independent experiments. Unpaired t test was conducted in (A) and (B), and one-way ANOVA was performed in (C), and paired *t*-test was used in (D).

One of the documented consequences of *C. trachomatis* infection in epithelial cells is the production of the pro-inflammatory cytokines IL6 and IL8 (32, 33). ISG15 is a negative regulator of IFN-I immunity, preventing over-amplification of inflammation (4). The observation that ISG15 was strongly induced during *Chlamydia* infection prompted us to test whether it modulated *Chlamydia*-induced inflammation. To this end, we silenced *ISG15* expression in HeLa cells using small-interfering RNA, which resulted in a strong reduction in ISG15 level, below detectable level (Fig. 2A, left panel). We observed an increase in the transcription levels of the two pro-inflammatory cytokine genes *IL6* and *IL8* upon silencing of *ISG15* (Fig. 2A, top panels). This was correlated with an increase in the amount of cytokine in the culture supernatant (Fig. 2A, bottom panels). In the rest of the manuscript, only transcriptional data are shown, as we consistently observed a good correlation between the two read-outs. We then used murine embryotic fibroblasts (MEFs) lacking ISG15 (8) to measure the innate response to infection. Consistent with what we had observed in human cells, mouse cells KO for *ISG15* displayed increased synthesis of IL6, and to a lesser extent of IL8 (KC), upon *Chlamydia* infection (Fig. 2B). Finally, we disrupted *ISG15* expression in HeLa cell using CRISPR/Cas9 and obtained four clones of homozygotes for *ISG15*, with a disruption of the open reading frame resulting in the absence of ISG15 expression (Fig. S1A). In three of them, we observed an increase in *IL6* and *IL8* transcriptions upon infection, compared with the parental wild-type cell line (Fig. S1B). This observation confirms that overall disruption of *ISG15* expression increases the expression of *IL6* and *IL8*, but that compensatory mechanisms may take place in some clones. Two clones C2 and C4 were selected for complementation experiments using the sleeping-beauty transposon system to achieve expression in the whole cell population (Fig. 2C, left panel; S1C) (34). Constitutive expression of ISG15 in C2 and C4 reduced basal and *Chlamydia*-induced transcription of *IL6*/*IL8* (Fig. 2C). Multiplex ELISA assay on culture supernatants confirmed that infection-stimulated secretion of IL6 and IL8, as well as of several other cytokines and chemokines, was reduced in cells expressing ISG15 compared with ISG15-KO HeLa cells (Fig. S2). The ISG15-sensitive secretome included pro-inflammatory cytokines/chemokines, anti-inflammatory cytokines, such as IL10 and IL13, and other actors of the immune response (e.g., TNFSF5, TNFSF10, FLT3L, granzyme B, etc). Altogether, these sets of data show that ISG15 normally acts as a brake on the host immune response to *C. trachomatis* infection.

### ISG15 expression restricts *C. trachomatis* development

We then investigated whether the absence of ISG15 expression affected *C. trachomatis* development. Wild-type and ISG15-KO HeLa cells were infected with *C. trachomatis* for 48 h (a duration corresponding on average to the completion of one developmental cycle) before measuring the progeny. We observed a moderate increase in infectious bacteria produced in the ISG15-KO background (1.8-fold for KO-C2 and 2.3-fold for KO-C4) compared with wild-type cells. A partial reversion of this effect was observed in the complemented cell lines (Fig. 2D). Consistently, we observed a small increase in the bacterial load upon silencing of *ISG15* in HeLa cells (1.3-fold) and in MEFs KO for *ISG15* (1.3-fold Fig. S3, left panels). The ability for the bacteria to attach to (Fig. S3, middle panels) and to penetrate into (Fig. S3, right panels) cells were not dependent on ISG15 expression in the two cellular backgrounds, indicating that the increase in bacterial load is linked to an increase in replication capacity, not in higher susceptibility to bacterial invasion. We concluded from these experiments that ISG15 expression restricted *C. trachomatis* development. The effect was however moderate and did probably not account for the strong increase in inflammatory cytokine production observed in the absence of ISG15 expression, as analysis of cytokine production at the single-cell level showed only a weak correlation between bacterial load and cytokine production (Fig. S4).

### IFN-I signaling does not account for ISG15 upregulation upon *Chlamydia* infection

**Fig 3:**
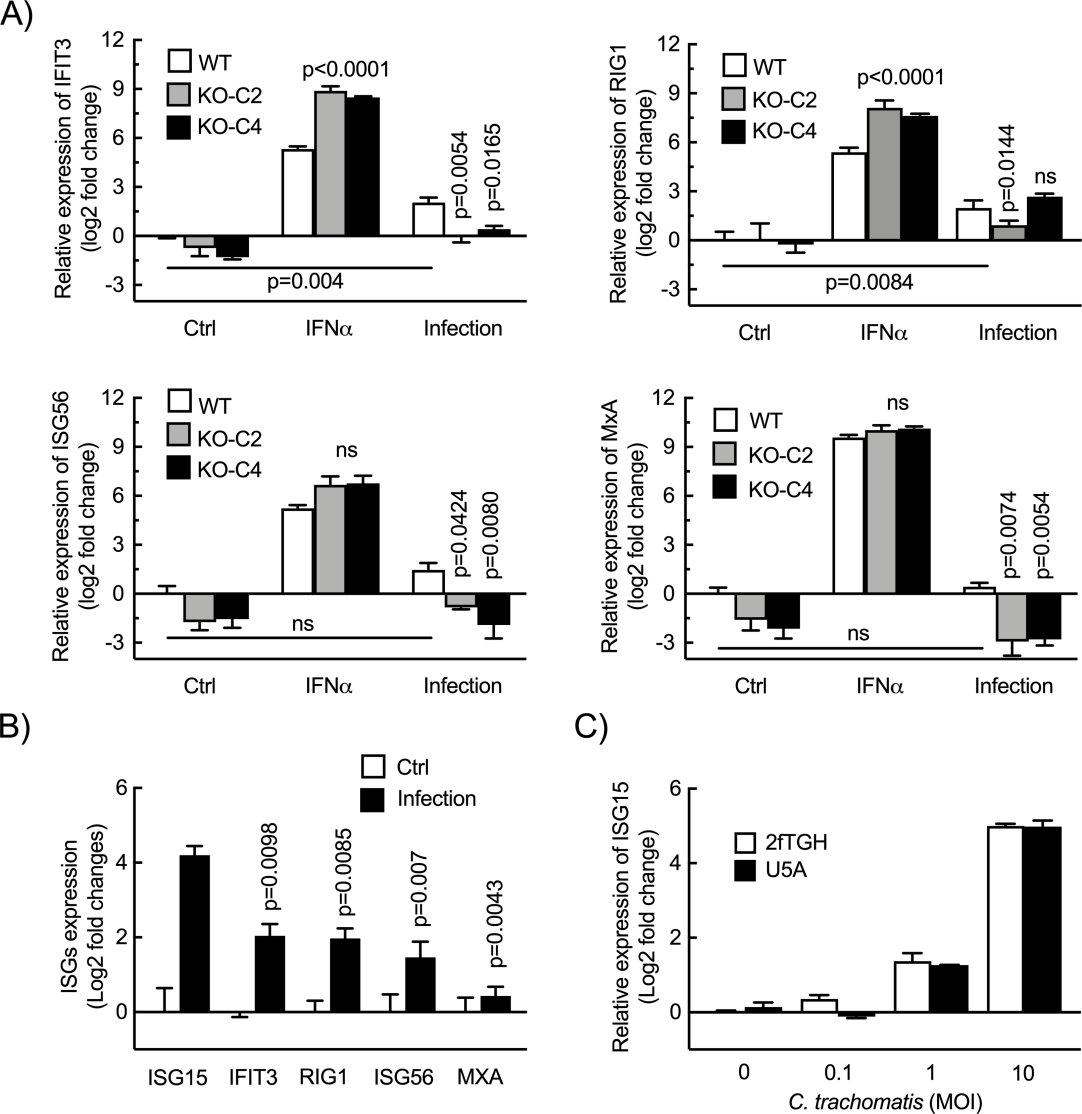
Autocrine IFN signaling is not implicated in *Chlamydia*-induced ISG15 expression by epithelial cells. (A) Two clones of ISG15-KO HeLa cells and wild-type cells were infected with *C. trachomatis* (MOI = 1) for 42 h or incubated with IFNα (10 ng/mL) for 24 h. After treatment, the transcript levels of the ISGs including *IFIT3*, *RIG1*, *ISG56,* and *MXA* normalized to *actin* were determined and expressed relative to non-infected WT cells. (**B**) HeLa cells were infected with *C. trachomatis* (MOI = 1) for 42 h. The expression of *ISGs* were normalized to *actin* and are expressed relative to non-infected cells. (**C**) U5A epithelial cells with mutated IFNa/b receptor and the parental 2fTGH cells were infected by *C. trachomatis* at the indicated MOI, and the *ISG15* transcript level normalized to *actin* was determined at 42 hpi. The data represent three independent experiments. Unpaired t test in A and one-way ANOVA test in B were performed. The p-values of the comparison with WT cells (**A**), *Chlamydia*-induced ISG15 (**B**), or as indicated are shown.

We next investigated the signaling pathway(s) leading to the transcriptional upregulation of *ISG15* expression during *C. trachomatis* infection. We first asked how specific the effect was on *ISG15* transcription as opposed to other ISGs. As expected, the transcript levels of four commonly studied ISGs, i.e., *IFIT3*, *RIG1*, *ISG56,* and *MXA*, were upregulated by IFNα. In the ISG15-KO HeLa cells IFNα stimulation resulted in a stronger transcriptional upregulation of these four genes compared with that observed in control cells (Fig. 3A), a phenomenon already documented (4). The transcription of these *ISGs* were also stimulated by *C. trachomatis* infection, but to a lesser extent than that of *ISG15* (Fig. 3B), and their induction was dependent on *ISG15* being present (Fig. 3A and B). This data indicated that, among ISGs, *ISG15* was particularly sensitive to the infectious context. Still, activation of several ISGs was consistent with the hypothesis that *ISG15* stimulation involved autocrine stimulation of cells by IFN-I. To test this hypothesis, we used U5A epithelial cells. These cells do not respond to IFN-I due to a mutation in the IFNα/b receptor (35). The upregulation in *ISG15* transcription in U5A cells upon *C. trachomatis* infection could not be distinguished from that in the parental 2fTGH cells (Fig. 3C). This result demonstrated that autocrine secretion of IFN-I did not account for the increase in *ISG15* transcription upon *Chlamydia* infection.

### ISG15 upregulation is not mediated by the PI3K/Akt and NF-κB signaling pathways

Given that *C. trachomatis* infection activates the PI3K/Akt signaling pathway (36), we then tested its implication in the upregulation of ISG15. Surprisingly, only low levels of Akt phosphorylation were detected upon *C. trachomatis* infection (Fig. S5A). Wortmannin, a PI3K inhibitor, had no apparent effect on *Chlamydia*-induced ISG15 expression (Fig. S5B, upper panel). As control, wortmannin abolished Akt basal level and phosphorylation induced by TNFα (Fig. S5B, lower panel). These data indicated that the PI3K/Akt signaling pathway was not implicated.

To test the contribution of the transcription factor NF-κB, we used HeLa cells stably expressing p65, a subunit of the most common p50/p65 heterodimer of NF-κB, as a GFP fusion protein under the control of the EF-1α promoter (37). TNFα stimulation was used as a positive control in these experiments. Treatment with TNFα triggered the translocation of p65-GFP to the nucleus, but *C. trachomatis* infection did not (Fig. S5C). These results indicated that the transcription factor NF-κB was not implicated in the upregulation of *ISG15* transcription upon *C. trachomatis* infection.

### ISG15 expression upon infection is triggered by activation of a cGAS/TBK1/STING/IRF3 pathway

**Fig 4:**
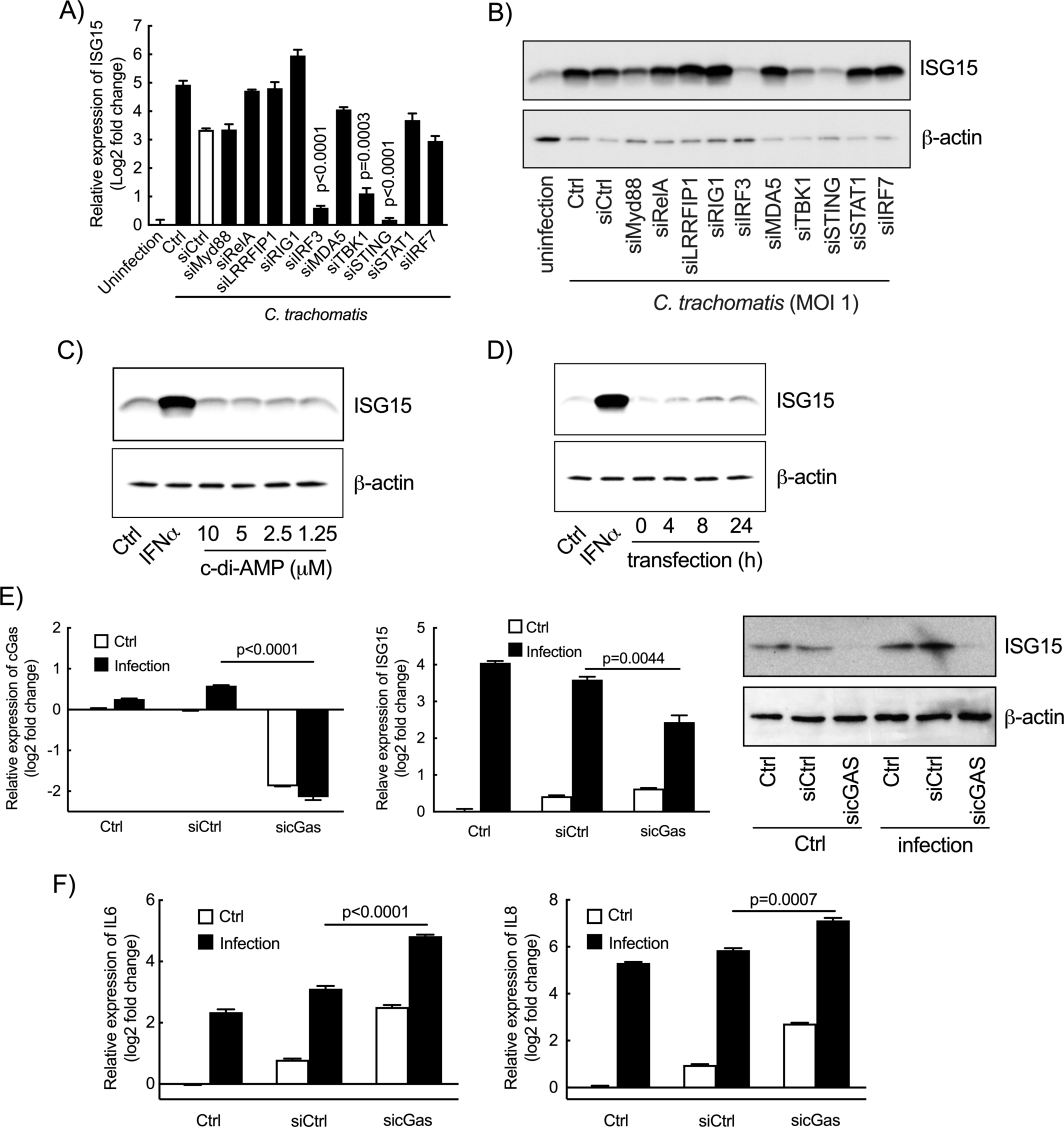
The TBK1/Sting/IRF3 pathway lies upstream of ISG15 synthesis by epithelial cells in response to *Chlamydia* infection. (**A-B**) The indicated genes were silenced by siRNA (30 nM) for 48 h before *Chlamydia* infection. At 42 h post-infection, ISG15 expression was examined either by RT-qPCR (**A**) or by immunoblot (**B**). (**C**) HeLa cells were incubated with c-di-AMP at the indicated concentration for 24 h before quantification of the ISG15 levels in whole cell lysates by Western blot. (**D**) c-di-AMP (10 µM) was introduced into HeLa cells using transfection reagent at 37°C for the indicated times. The cells were then washed and were incubated for an additional 24 h before examining ISG15 level in whole cell lysates by Western blot. Incubation of HeLa cells with IFNα (10 ng/mL) for 24 h was used as positive control (C and D). (**E-F**) siRNA against *cGAS* or irrelevant oligonucleotides were transfected into HeLa cells for 48 h before *Chlamydia* infection. At 42 h post-infection, the expression of *cGAS* (E, left panel), *ISG15* (E, middle and right panels), *IL6* and *IL8* (**F**), and *actin* were measured. All data represent three independent experiments. The p-values of unpaired t test with the condition “control siRNA” (**A**) or between the indicated conditions (**E, F**) are shown.

We next screened several transcriptional factors or mediators of various signaling pathways for their contribution to *ISG15* up-regulation, i.e., Myd88, RelA, LRRFIP1, RIG1, IRF3, MDA5, TBK1, STING, STAT1, and IRF7. Two days after silencing, the cells were infected with *C. trachomatis*. The cell lysates were collected 42 h later to measure *ISG15* transcripts and protein levels. *IRF3*, *TBK1,* and *STING* were the only three genes whose silencing markedly decreased *Chlamydia*-induced *ISG15* transcription in HeLa cells (Fig. 4A). Consistently, their silencing also prevented the increase in ISG15 levels measured by Western blot (Fig. 4B). These three genes participate in the same signaling pathways, as STING (stimulator of interferon genes) and the kinase TBK1 interact with each other, and are essential for the activation of IFN-I genes through subsequent activation of the transcription factor IRF3 (38, 39). To confirm their implication in ISG15 upregulation, we tested the effect of the STING activator, c-di-AMP, on ISG15 synthesis. c-di-AMP (BioLog Life Sciences, #C088-01) had no effect on ISG15 expression by HeLa cells when applied in the culture medium, even at a concentration of 10 µM (Fig. 4C). In contrast, introducing c-di-AMP into HeLa cells using transfection reagents stimulated ISG15 expression (Fig. 4D). STING can also be activated by cyclic GMP-AMP, a unique secondary messenger synthesized from GTP and ATP by the cytosolic GMP-AMP synthase (cGAS) (40). To test the role of cGAS in ISG15 induction upon *Chlamydia* infection, we silenced the expression of this enzyme in HeLa cells before infection. We observed a significant decrease in the amount of *ISG15* transcripts and ISG15 protein (Fig. 4E), indicating that ISG15 is produced in response to cytosolic DNA detection by the cGAS/STING pathway. We also observed an increase in the transcription of *IL6* and *IL8* genes upon cGAS silencing (Fig. 4F). This result is fully consistent with a need for cGAS to induce ISG15 expression during infection, and thereby dampen the expression of inflammatory genes. Thus, these experiments position *ISG15* transcription downstream of the activation of the c-di-AMP/TBK1/STING/IRF3 and cGAS/TBK1/STING/IRF3 pathways upon *C. trachomatis* infection.

### The ISG15 brake on inflammation during *Chlamydia* infection acts intracellularly independently of ISGylation

**Fig 5:**
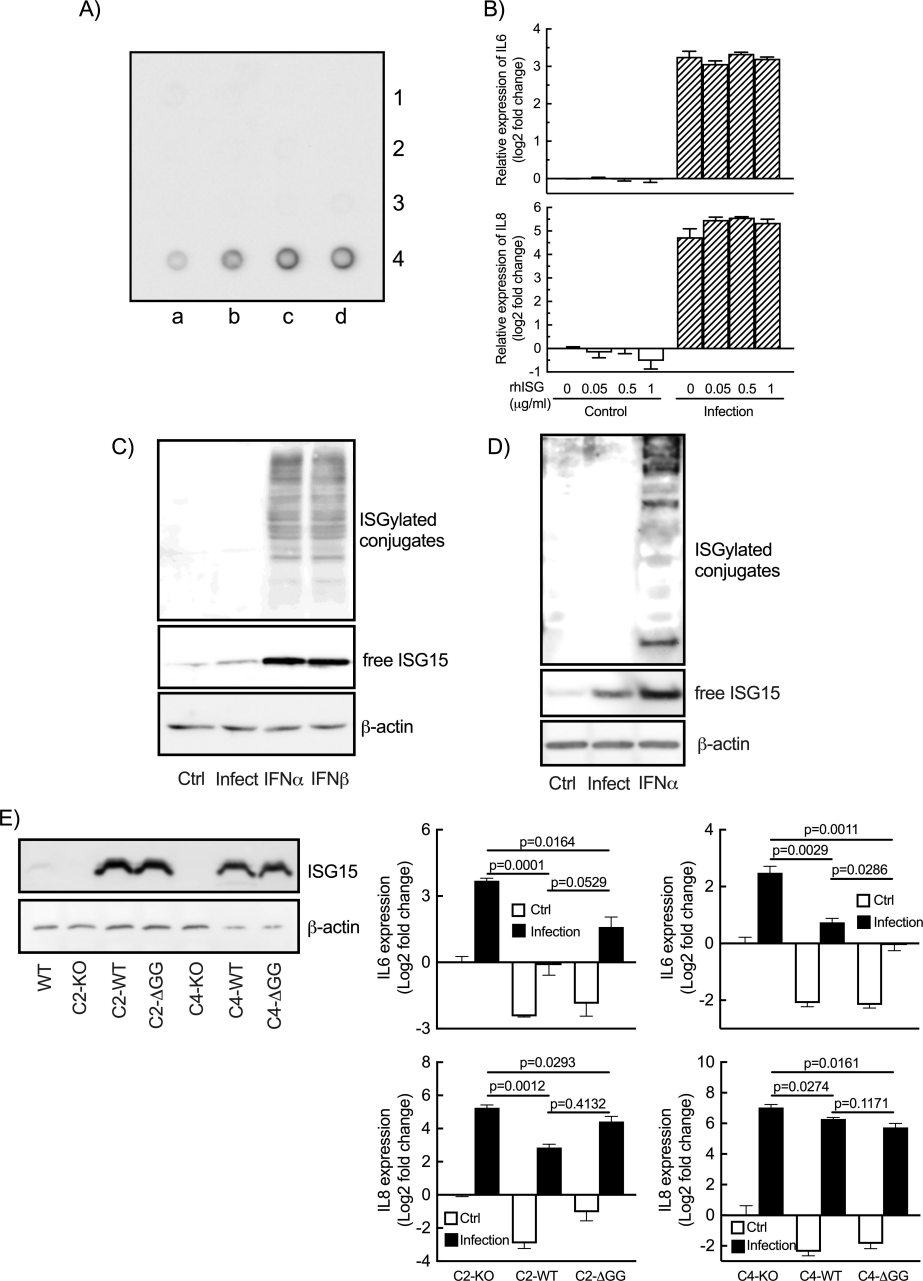
ISG15 regulates inflammation from an intracellular location and independently of ISGylation. (**A**) Culture medium was collected at different times post-infection for ISG15 detection by dot blot. First row: 0, 3, 6, and 9 hpi; second row: 12, 18, 24, and 30 hpi; third row: 36, 42, and 48 hpi; fourth row: 25, 50, 100, and 200 ng/mL of rhISG15. (**B**) ISG15-KO HeLa cells were pre-treated with the indicated concentration of rhISG15 for 30 min before *Chlamydia* infection (MOI = 1). The cells were further incubated for 42 h in the presence of rhISG15 before RNA extraction and quantification of *IL6* and *IL8* expression by RT-qPCR. (**C**) HeLa cells were incubated with *C. trachomatis* (MOI = 1) for 42 h or with IFN-α/β (10 ng/mL) for 24 h, lysed, and the amount of ISG15 (free form & conjugates) was analyzed by immunoblot on whole cell lysates. (**D**) Primary cervical epithelial cells were treated with IFNα (10 ng/mL) for 24 h or with *C. trachomatis* (MOI = 10) for 42 h followed by ISG15 detection. Top panels of C and D show overexposed images of the upper part of the membranes to visualize ISGylated proteins. (**E**) ISG15-KO cells (C2 and C4) or complemented cells (with ISG15 WT or ΔGG) were infected or not with *C. trachomatis* (MOI = 1) for 42 h before measuring the transcription of *IL6* and *IL8*. The panel on the left shows ISG15 expression in the complemented cells. The images displayed are representative of two independent experiments in A and of three independent experiments in D using primary cells from three individuals. All other data correspond to three independent experiments. The p-values of unpaired t test between the indicated groups are shown.

ISG15 is a cytoplasmic molecule that is also secreted by multiple cell types, including neutrophils, epithelial cells, monocytes, and T cells (6, 41). The extracellular form exerts biological functions, and its receptor has been identified (3). We thus tested the possibility that *Chlamydia*-induced ISG15 was secreted extracellularly and controlled the expression of proinflammatory cytokines by HeLa cells upon bacterial infection from this location. First, the culture medium of infected HeLa cells was collected to probe for the presence of secreted ISG15 by dot blot. Assays using serial quantities of recombinant human ISG15 (rhISG15, R&D systems) determined that the sensitivity of the technique was sufficient to detect up to 25 ng/mL rhISG15 (Fig. 5A). No signal was detected when the membrane was probed with culture medium from infected wells (Fig. 5A), indicating that extracellular ISG15 concentration is lower than this threshold. Furthermore, incubation of ISG15-KO HeLa cells with up to 1 μg/mL rhISG15 had no effect on the basal, nor on the infection-induced, levels of *IL6* and *IL8* transcripts (Fig. 5B). These results demonstrate that extracellular ISG15 does not affect *Chlamydia*-induced inflammation, indicating that it exerts this activity inside the cell.

Intracellular ISG15 displays biological activities either as a free molecule or through conjugation to other proteins, a process called ISGylation (41, 42). We tested whether *Chlamydia* infection was accompanied with global changes in the level of ISGylation. IFNα and β were used as positive controls. As expected, HeLa cell exposure to these cytokines elicited a strong increase in ISG15 expression and protein ISGylation (Fig. 5C). Infection induced ISG15 expression, but to a lower extent than IFN-I did, and protein ISGylation was below detection limit (Fig. 5C). Similar results were obtained in *Chlamydia*-infected primary cervical epithelial cells of patients (Fig. 5D). To determine whether ISGylation was implicated in the ISG15-mediated restriction on inflammation, we complemented two ISG15-KO clones with truncated ISG15-ΔGG, that is not competent for ISGylation (43). We observed that expression of ISG15-ΔGG, attenuated *Chlamydia*-induced expression of *IL6* and *IL8* to the same extent as ISG15-WT (Fig. 5E). These experiments demonstrate that the ability for ISG15 to dampen inflammation does not involve ISGylation.

### Lack of ISG15 results in exacerbated tissue damage in a mouse model of infection

**Fig 6:**
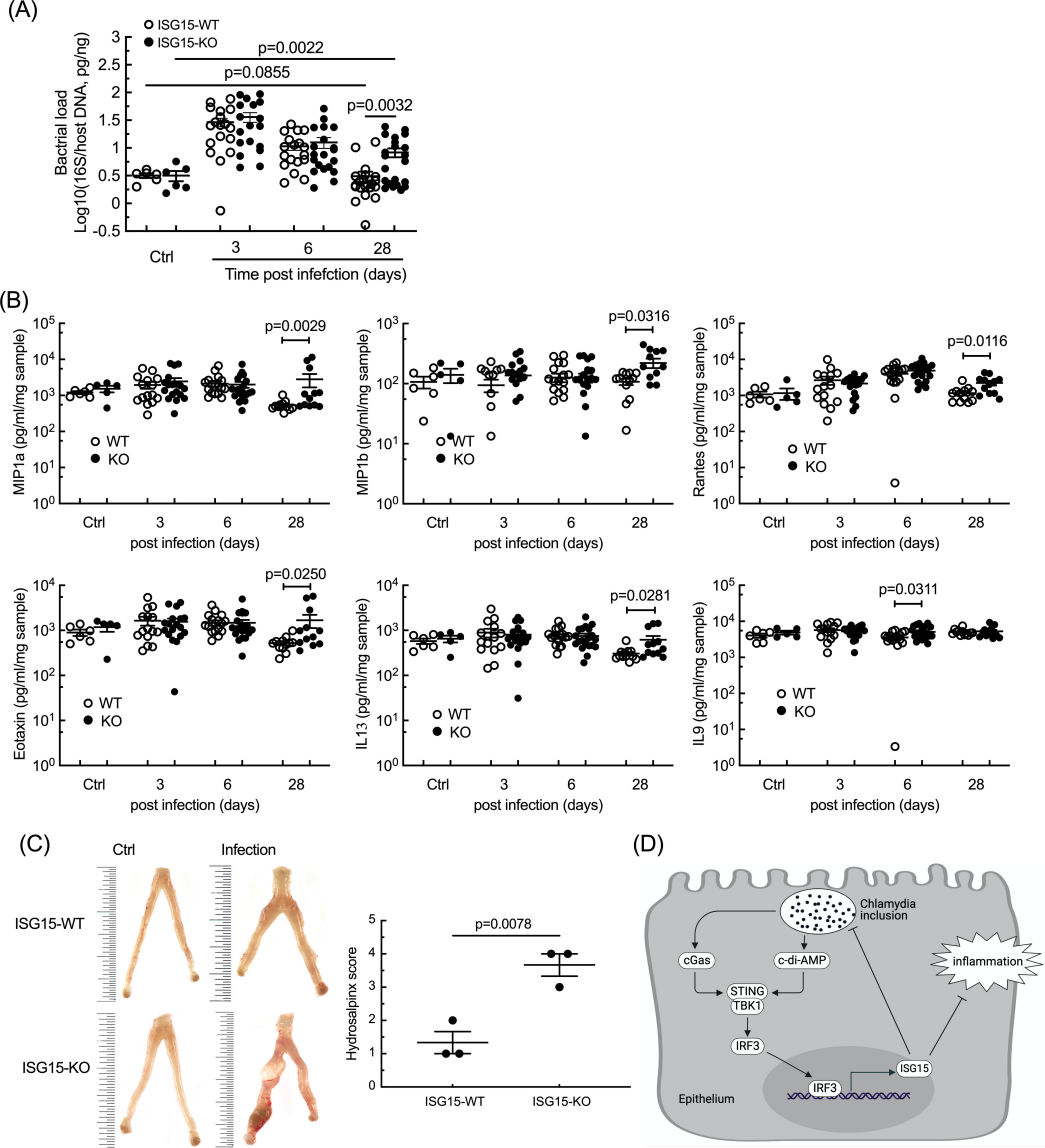
ISG15 limits the inflammatory response in the upper FGT of mice infected with *C. trachomatis*. ISG15-KO and ISG15-WT mice were infected by introducing *C. trachomatis* into the uterine horn trans-cervically. The upper FGTs were collected at the indicated times post-infection. The upper FGTs from non-infected mice were collected as negative control. Bacterial loads (**A**) and inflammatory cytokines (**B**) in FGT homogenates were determined using quantitative PCR and multi-plex ELISA assays, respectively. Each dot represents one animal. (**C**) Representative FGTs (left) and hydrosalpinx index for six infected animals (right). The smallest interval of the scale in this image represents 0.05 cm. The p-value of unpaired t test between indicated groups are shown. (**D**) Graphical summary. *Chlamydia* infection of epithelial cells is sensed by cGas, which activates the STING/TBK1/IRF3 signaling cascade. Direct release of c-di-AMP by the bacteria may also be involved. IRF3 activates transcription of *ISG15*. Increase in intracellular free ISG15 restricts bacterial proliferation and the transcription of the host inflammatory cytokines IL6 and IL8, independently of ISGylation.

We finally tested the consequence of ISG15 deficiency using a trans-cervical mouse infection model for *C. trachomatis* infection (30). Bacterial loads measured on days 3 and 6 were not different between the two groups of mice. At 4 weeks post-infection, infection was cleared in almost all WT mice (Fig. 6A). However, bacterial DNA was still detected in about half of the ISG15-KO mice (Fig. 6A), and higher levels for several proinflammatory cytokines in the genital tract of ISG15-KO animals were observed, i.e., MIP1α, MIP1β, Rantes, IL13, and Eotaxin (Fig. 6B; Fig. S6). ISG15-KO animals also displayed more severe signs of hydrosalpinx compared with wild-type mice (Fig. 6C). Thus, ISG15 deficiency weakened the natural ability of mice to clear *C. trachomatis* infection, and exacerbated inflammation and subsequent tissue damage.

## DISCUSSION

The lower FGT of women is exposed to the vaginal microbiota, and occasionally to a variety of sexually transmitted pathogens, including HPV, HIV, and *C. trachomatis*. The latter induces an inflammatory response aiming at the elimination of the bacteria, but which is also at the root of the pathological consequences of *Chlamydia* infection. In this report, we show that infection of primary cervical epithelial cells, as well as of the HeLa cell line, by *C. trachomatis* elicits a strong induction of the expression of the ubiquitin-like molecule ISG15, and that this molecule acts as a brake on the immune response to infection. Reducing or knocking-out the expression of *ISG15*, in different cellular backgrounds, resulted in an increase in the transcription and secretion of two inflammatory cytokines, IL6 and IL8, which were rescued by ISG15 complementation into ISG15-KO cells. Quantification of cytokines and chemokines by ELISA confirmed that ISG15 broadly repressed the immune response to infection. As discussed below, we describe the signaling pathway that triggers ISG15 expression. Overall, the induction of ISG15 in epithelial cells upon *C. trachomatis* infection benefits the host by limiting bacterial replication and circumventing the inflammatory response (Fig. 6D).

Induction of ISG15 expression upon *C. trachomatis* infection was observed in HeLa cells, in primary cells, in MEFs (this report), and in a fallopian tube-derived organoid model (44). We showed that silencing the TBK1/STING/IRF3 signaling cascade was sufficient to prevent *ISG15* induction upon *C. trachomatis* infection. During viral infection, activation of the TBK1/STING/IRF3 signaling cascade leads to the expression of IFN-I (38, 39). IFN-I binding to its receptor IFNAR triggers the canonical STAT signaling cascade, resulting in the transcription of many IFN-responsive genes, including *ISG15*. Interestingly, in the infection situation studied here, *ISG15* transcription was induced even in the absence of IFNAR signaling (Fig. 3C). The increase in ISG15 level upon another bacterial infection, *Listeria monocytogenes*, was also independent of IFN-I (8). It is important to note that the increase in ISG15 upon *C. trachomatis* infection was lower than upon IFN-I induction (Fig. 1D, E, 5C and D). Therefore, the amplification of *ISG15* transcription upon IFN-I signaling may mask the intrinsic capacity of the TBK1/STING/IRF3 signaling cascade to directly upregulate *ISG15* and account for the apparent difference between viral and bacterial infections regarding the regulation of *ISG15* transcription. In other words, the low level of IFN-I induction in epithelial cells infected by *Chlamydia* enables to detect the direct regulation of *ISG15* expression by the TBK1/STING/IRF3 signaling cascade.

Silencing cGAS resulted in a strong decrease in the *Chlamydia*-induced upregulation of ISG15, indicating that detection of nucleic acids was involved, such as in viral infection. This is consistent with the observation that c-di-AMP is a prominent ligand for STING-mediated activation of IFN responses during *C. trachomatis* infection (45). Interestingly, *C. trachomatis* possesses an enzyme that synthesizes c-di-AMP (45). This might contribute to direct STING activation, in addition to cGAS generated cyclic GMP-AMP. In contrast, *Chlamydia*-induced IL1β in macrophages was dependent of STING but independent of cGAS (46).

Silencing, or knocking out, the expression of ISG15 enhanced the expression of the inflammatory cytokines IL6 and IL8, which was rescued by complementation with *ISG15* under a constitutive promoter (Fig. 2C and 5E), indicating that ISG15 acts as a negative regulator of inflammation. Consistent with this hypothesis, the transcriptional induction of *IL6* and *IL8* starts late in HeLa cells, when each vacuole contains already several hundreds of bacteria (47, 48). In contrast, *ISG15* transcription is induced within 6 h of infection. These kinetics are consistent with a scenario in which ISG15 attenuates the induction of the pro-inflammatory signals. On the contrary, overexpression of human ISG15 enhanced IL6 and IL8 production induced by *L. monocytogenes* infection in HeLa cells (8).

Mechanistically, we uncovered another important difference when comparing *Chlamydia* to other bacterial infections. Both *Listeria* and *Mycobaterium* have been shown to elicit ISGylation *in vitro* and *in vivo*, respectively (8, 49). In contrast, in spite of the increase in free ISG15, ISGylated proteins remained below detection threshold by Western blot in *C. trachomatis*-infected cells (Fig. 5C and D). This was not due to an increase in USP18, a protease reversing ISGylation (50), since the protease level was stable over the course of infection (data not shown). *C. trachomatis* secretes two proteins, one with deubiquitinase and acyltransferase activities, the second with deubiquitinase activity (51, 52). These enzymes did not utilize ISG15 to form conjugates *in vitro*, indicating that they are not responsible for the low level of ISGylation in *C. trachomatis*-infected cells (51). Whether another secreted bacterial factor limits ISGylation remains to be explored. In any case, we showed here that ISGylation was dispensable for the anti-inflammatory activity of ISG15 in epithelial cells, and that this activity was exerted intracellularly. The mechanism(s) by which free ISG15 level modulate the transcription of *IL6* and *IL8* require further investigation.

The ISGylation-independent control on IFN-I signaling during viral infection was described to operate in human, not in mouse, cells (7). Nevertheless, we decided to test the course of infection by *C. trachomatis* in a mouse model of infection for three reasons : (i) we had observed that in the case of *C. trachomatis* infection, mouse and human epithelial cells showed consistent phenotypes, i.e., a slightly increased bacterial proliferation (Fig. 2D; Fig. S3B) and a stronger proinflammatory signaling (Fig. 2) in the absence of ISG15; (ii) ISG15 is crucial for IFN-γ-dependent antimycobacterial immunity also in mice (6), and IFN-γ plays a key role in the early clearance of *C. trachomatis* infection in mice (30); (iii) only a few non-viral infections have been tested in ISG15 KO mice yet. We observed no difference in bacterial load at 3 and 6 days of infection between wild-type and ISG15 KO mice (Fig. 6A). Similar levels of IFN-γ were detected in ISG15-WT and ISG15-KO mice (Fig. S6). This set of data suggests that, in the context of *C. trachomatis* infection, ISG15 only plays a marginal role, if any, on regulating IFN-γ levels in mice. Still, ISG15 had a beneficial role in this mouse model of *C. trachomatis* infection, as its absence resulted in delayed bacterial clearance and increased tissue damage. We showed that its activity on limiting the immune response of human epithelial cells was exerted as a free intracellular molecule. It is possible that other modes of actions contribute to harnessing the immune response to *C. trachomatis* infection in mice, with different modes of action depending on the cell types.

## SUPPLEMENTAL MATERIAL

Figure S1 mbio.02401-24-s0001.tif Characterization of ISG15-KO cells.

Figure S2 mbio.02401-24-s0002.pdf ISG15 dampens the host immune response to *C. trachomatis* infection.

Figure S3 mbio.02401-24-s0003.tiff ISG15 depletion favors bacterial growth.

Figure S4 mbio.02401-24-s0004.tiff Single-cell analysis of bacterial load and cytokine production.

Figure S5 mbio.02401-24-s0005.pdf PI3K/Akt and NF-κB signaling pathways are not involved in *Chlamydia*-induced ISG15 synthesis by epithelial cells.

Figure S6 mbio.02401-24-s0006.tiff Absence of ISG15 delays *C. trachomatis* clearance and exacerbates tissue damage.

Legends mbio.02401-24-s0007.docx Legends for supplemental figures.

Table S1 mbio.02401-24-s0008.docx Primers.

ASM does not own the copyrights to Supplemental Material that may be linked to, or accessed through, an article. The authors have granted ASM a non-exclusive, world-wide license to publish the Supplemental Material files. Please contact the corresponding author directly for reuse.

## References

[1] Knight E, Fahey D, Cordova B, Hillman M, Kutny R, Reich N, Blomstrom D. 1988. A 15-kDa interferon-induced protein is derived by COOH-terminal processing of A 17-kDa precursor. J Biol Chem 263:4520–4522. 3350799

[2] Bogunovic D, Boisson-Dupuis S, Casanova JL. 2013. ISG15: leading a double life as a secreted molecule. Exp Mol Med 45:e18. doi:10.1038/emm.2013.36 23579383 PMC3641400

[3] Swaim CD, Scott AF, Canadeo LA, Huibregtse JM. 2017. Extracellular ISG15 signals cytokine secretion through the LFA-1 integrin receptor. Mol Cell 68:581–590. doi:10.1016/j.molcel.2017.10.003 29100055 PMC5690536

[4] Zhang X, Bogunovic D, Payelle-Brogard B, Francois-Newton V, Speer SD, Yuan C, Volpi S, Li Z, Sanal O, Mansouri D, et al.. 2015. Human intracellular ISG15 prevents interferon-α/β over-amplification and auto-inflammation. Nature New Biol 517:89–93. doi:10.1038/nature13801 PMC4303590 25307056

[5] Perng YC, Lenschow DJ. 2018. ISG15 in antiviral immunity and beyond. Nat Rev Microbiol 16:423–439. doi:10.1038/s41579-018-0020-5 29769653 PMC7097117

[6] Bogunovic D, Byun M, Durfee LA, Abhyankar A, Sanal O, Mansouri D, Salem S, Radovanovic I, Grant AV, Adimi P, et al.. 2012. Mycobacterial disease and impaired IFN-γ immunity in humans with inherited ISG15 deficiency. Science 337:1684–1688. doi:10.1126/science.1224026 22859821 PMC3507439

[7] Speer SD, Li Z, Buta S, Payelle-Brogard B, Qian L, Vigant F, Rubino E, Gardner TJ, Wedeking T, Hermann M, et al.. 2016. ISG15 deficiency and increased viral resistance in humans but not mice. Nat Commun 7:11496. doi:10.1038/ncomms11496 27193971 PMC4873964

[8] Radoshevich L, Impens F, Ribet D, Quereda JJ, Nam Tham T, Nahori M-A, Bierne H, Dussurget O, Pizarro-Cerdá J, Knobeloch K-P, Cossart P. 2015. ISG15 counteracts Listeria monocytogenes infection. Elife 4:e06848. doi:10.7554/eLife.06848 26259872 PMC4530601

[9] WHO. 2023. Sexually transmitted infections (STIs). Available from: https://www.who.int/news-room/fact-sheets/detail/sexually-transmitted-infections-(stis)

[10] Jasumback CL, Perry SH, Ness TE, Matsenjwa M, Masangane ZT, Mavimbela M, Mthethwa N, Dlamini L, Mphaya J, Kirchner HL, Mandalakas A, Kay AW. 2020. Point-of-care testing to guide treatment and estimate risk factors for sexually transmitted infections in adolescents and young people with human immunodeficiency virus in eswatini. Open Forum Infect Dis 7:faa052. doi:10.1093/ofid/ofaa052 PMC7071112 32190707

[11] Sturdevant GL, Caldwell HD. 2014. Innate immunity is sufficient for the clearance of Chlamydia trachomatis from the female mouse genital tract. Pathog Dis 72:70–73. doi:10.1111/2049-632X.12164 24585717 PMC4152394

[12] Wong WF, Chambers JP, Gupta R, Arulanandam BP. 2019. Chlamydia and its many ways of escaping the host immune system. J Pathog 2019:8604958. doi:10.1155/2019/8604958 31467721 PMC6699355

[13] Rajeeve K, Das S, Prusty BK, Rudel T. 2018. Chlamydia trachomatis paralyses neutrophils to evade the host innate immune response. Nat Microbiol 3:824–835. doi:10.1038/s41564-018-0182-y 29946164

[14] Tang L, Chen J, Zhou Z, Yu P, Yang Z, Zhong G. 2015. Chlamydia-secreted protease CPAF degrades host antimicrobial peptides. Microbes Infect 17:402–408. doi:10.1016/j.micinf.2015.02.005 25752416

[15] Patton MJ, McCorrister S, Grant C, Westmacott G, Fariss R, Hu P, Zhao K, Blake M, Whitmire B, Yang C, Caldwell HD, McClarty G. 2016. Chlamydial protease-like activity factor and type III secreted effectors cooperate in inhibition of p65 nuclear translocation. mBio 7:e01427-16. doi:10.1128/mBio.01427-16 27677792 PMC5040114

[16] Haldar AK, Piro AS, Finethy R, Espenschied ST, Brown HE, Giebel AM, Frickel EM, Nelson DE, Coers J. 2016. Chlamydia trachomatis is resistant to inclusion ubiquitination and associated host defense in gamma interferon-primed human epithelial cells. mBio 7:e01417-16. doi:10.1128/mBio.01417-16 27965446 PMC5156299

[17] Carpenter V, Chen YS, Dolat L, Valdivia RH. 2017. The effector TepP mediates recruitment and activation of phosphoinositide 3-kinase on early Chlamydia trachomatis vacuoles. mSphere 2:e00207-17 doi:10.1128/mSphere.00207-17 28744480 PMC5518268

[18] Chen YS, Bastidas RJ, Saka HA, Carpenter VK, Richards KL, Plano GV, Valdivia RH. 2014. The Chlamydia trachomatis type III secretion chaperone Slc1 engages multiple early effectors, including TepP, a tyrosine-phosphorylated protein required for the recruitment of CrkI-II to nascent inclusions and innate immune signaling. PLoS Pathog 10:e1003954. doi:10.1371/journal.ppat.1003954 24586162 PMC3930595

[19] Pierangeli A, Degener AM, Ferreri ML, Riva E, Rizzo B, Turriziani O, Luciani S, Scagnolari C, Antonelli G. 2011. Interferon-induced gene expression in cervical mucosa during human papillomavirus infection. Int J Immunopathol Pharmacol 24:217–223. doi:10.1177/039463201102400126 21496405

[20] Cotter TW, Ramsey KH, Miranpuri GS, Poulsen CE, Byrne GI. 1997. Dissemination of Chlamydia trachomatis chronic genital tract infection in gamma interferon gene knockout mice. Infect Immun 65:2145–2152. doi:10.1128/iai.65.6.2145-2152.1997 9169744 PMC175296

[21] Ito JI, Lyons JM. 1999. Role of gamma interferon in controlling murine chlamydial genital tract infection. Infect Immun 67:5518–5521. doi:10.1128/IAI.67.10.5518-5521.1999 10496942 PMC96917

[22] Lampe MF, Wilson CB, Bevan MJ, Starnbach MN. 1998. Gamma interferon production by cytotoxic T lymphocytes is required for resolution of Chlamydia trachomatis infection. Infect Immun 66:5457–5461. doi:10.1128/IAI.66.11.5457-5461.1998 9784557 PMC108683

[23] Tang C, Liu C, Maffei B, Niragire B, Cohen H, Kane A, Donnadieu AC, Levy-Zauberman Y, Vernay T, Hugueny J, Vincens E, Louis-Sylvestre C, Subtil A, Wu Y. 2021. Primary ectocervical epithelial cells display lower permissivity to Chlamydia trachomatis than HeLa cells and a globally higher pro-inflammatory profile. Sci Rep 11:5848. doi:10.1038/s41598-021-85123-7 33712643 PMC7955086

[24] Ran FA, Hsu PD, Wright J, Agarwala V, Scott DA, Zhang F. 2013. Genome engineering using the CRISPR-Cas9 system. Nat Protoc 8:2281–2308. doi:10.1038/nprot.2013.143 24157548 PMC3969860

[25] Vromman F, Laverrière M, Perrinet S, Dufour A, Subtil A. 2014. Quantitative monitoring of the Chlamydia trachomatis developmental cycle using GFP-expressing bacteria, microscopy and flow cytometry. PLoS One 9:e99197. doi:10.1371/journal.pone.0099197 24911516 PMC4049595

[26] Agaisse H, Derré I. 2013. A C. trachomatis cloning vector and the generation of C. trachomatis strains expressing fluorescent proteins under the control of A C. trachomatis promoter. PLoS One 8:e57090. doi:10.1371/journal.pone.0057090 23441233 PMC3575495

[27] Scidmore MA. 2005. Cultivation and laboratory maintenance of Chlamydia trachomatis. Curr Protoc Microbiol Chapter 11:Unit 11A.1. doi:10.1002/9780471729259.mc11a01s00 18770550

[28] Chen RH, Du Y, Han P, Wang HB, Liang FY, Feng GK, Zhou AJ, Cai MY, Zhong Q, Zeng MS, Huang XM. 2016. ISG15 predicts poor prognosis and promotes cancer stem cell phenotype in nasopharyngeal carcinoma. Oncotarget 7:16910–16922. doi:10.18632/oncotarget.7626 26919245 PMC4941359

[29] Livak KJ, Schmittgen TD. 2001. Analysis of relative gene expression data using real-time quantitative PCR and the 2(-Delta Delta C(T)) method. Methods 25:402–408. doi:10.1006/meth.2001.1262 11846609

[30] Gondek DC, Olive AJ, Stary G, Starnbach MN. 2012. CD4+ T cells are necessary and sufficient to confer protection against Chlamydia trachomatis infection in the murine upper genital tract. J Immunol 189:2441–2449. doi:10.4049/jimmunol.1103032 22855710 PMC3690950

[31] Peng B, Lu C, Tang L, Yeh IT, He Z, Wu Y, Zhong G. 2011. Enhanced upper genital tract pathologies by blocking Tim-3 and PD-L1 signaling pathways in mice intravaginally infected with Chlamydia muridarum. BMC Infect Dis 11:347. doi:10.1186/1471-2334-11-347 22168579 PMC3259114

[32] Cunningham K, Stansfield SH, Patel P, Menon S, Kienzle V, Allan JA, Huston WM. 2013. The IL-6 response to Chlamydia from primary reproductive epithelial cells is highly variable and may be involved in differential susceptibility to the immunopathological consequences of chlamydial infection. BMC Immunol 14:50. doi:10.1186/1471-2172-14-50 24238294 PMC4225670

[33] Rasmussen SJ, Eckmann L, Quayle AJ, Shen L, Zhang YX, Anderson DJ, Fierer J, Stephens RS, Kagnoff MF. 1997. Secretion of proinflammatory cytokines by epithelial cells in response to Chlamydia infection suggests a central role for epithelial cells in chlamydial pathogenesis. J Clin Invest 99:77–87. doi:10.1172/JCI119136 9011579 PMC507770

[34] Kowarz E, Löscher D, Marschalek R. 2015. Optimized sleeping beauty transposons rapidly generate stable transgenic cell lines. Biotechnol J 10:647–653. doi:10.1002/biot.201400821 25650551

[35] Lutfalla G, Holland SJ, Cinato E, Monneron D, Reboul J, Rogers NC, Smith JM, Stark GR, Gardiner K, Mogensen KE. 1995. Mutant U5A cells are complemented by an interferon-alpha beta receptor subunit generated by alternative processing of a new member of a cytokine receptor gene cluster. EMBO J 14:5100–5108. doi:10.1002/j.1460-2075.1995.tb00192.x 7588638 PMC394613

[36] Verbeke P, Welter-Stahl L, Ying S, Hansen J, Häcker G, Darville T, Ojcius DM. 2006. Recruitment of BAD by the Chlamydia trachomatis vacuole correlates with host-cell survival. PLoS Pathog 2:e45. doi:10.1371/journal.ppat.0020045 16710454 PMC1463014

[37] Connor MG, Camarasa TMN, Patey E, Rasid O, Barrio L, Weight CM, Miller DP, Heyderman RS, Lamont RJ, Enninga J, Hamon MA. 2021. The histone demethylase KDM6B fine-tunes the host response to Streptococcus pneumoniae. Nat Microbiol 6:257–269. doi:10.1038/s41564-020-00805-8 33349663

[38] Orzalli MH, DeLuca NA, Knipe DM. 2012. Nuclear IFI16 induction of IRF-3 signaling during herpesviral infection and degradation of IFI16 by the viral ICP0 protein. Proc Natl Acad Sci U S A 109:E3008–17. doi:10.1073/pnas.1211302109 23027953 PMC3497734

[39] Tanaka Y, Chen ZJ. 2012. STING specifies IRF3 phosphorylation by TBK1 in the cytosolic DNA signaling pathway. Sci Signal 5:ra20. doi:10.1126/scisignal.2002521 22394562 PMC3549669

[40] Motwani M, Pesiridis S, Fitzgerald KA. 2019. DNA sensing by the cGAS-STING pathway in health and disease. Nat Rev Genet 20:657–674. doi:10.1038/s41576-019-0151-1 31358977

[41] Dos Santos PF, Mansur DS. 2017. Beyond ISGlylation: functions of free Intracellular and extracellular ISG15. J Interferon Cytokine Res 37:246–253. doi:10.1089/jir.2016.0103 28467275

[42] Dzimianski JV, Scholte FEM, Bergeron É, Pegan SD. 2019. ISG15: it’s complicated. J Mol Biol 431:4203–4216. doi:10.1016/j.jmb.2019.03.013 30890331 PMC6746611

[43] Bade VN, Nickels J, Keusekotten K, Praefcke GJK. 2012. Covalent protein modification with ISG15 via a conserved cysteine in the hinge region. PLoS One 7:e38294. doi:10.1371/journal.pone.0038294 22693631 PMC3367918

[44] Kessler M, Hoffmann K, Fritsche K, Brinkmann V, Mollenkopf H-J, Thieck O, Teixeira da Costa AR, Braicu EI, Sehouli J, Mangler M, Berger H, Meyer TF. 2019. Chronic Chlamydia infection in human organoids increases stemness and promotes age-dependent CpG methylation. Nat Commun 10:1194. doi:10.1038/s41467-019-09144-7 30886143 PMC6423033

[45] Barker JR, Koestler BJ, Carpenter VK, Burdette DL, Waters CM, Vance RE, Valdivia RH. 2013. STING-dependent recognition of cyclic di-AMP mediates type I interferon responses during Chlamydia trachomatis infection. mBio 4:e00018-13. doi:10.1128/mBio.00018-13 23631912 PMC3663186

[46] Webster SJ, Brode S, Ellis L, Fitzmaurice TJ, Elder MJ, Gekara NO, Tourlomousis P, Bryant C, Clare S, Chee R, Gaston HJS, Goodall JC. 2017. Detection of a microbial metabolite by STING regulates inflammasome activation in response to Chlamydia trachomatis infection. PLoS Pathog 13:e1006383. doi:10.1371/journal.ppat.1006383 28570638 PMC5453623

[47] Buchholz KR, Stephens RS. 2006. Activation of the host cell proinflammatory interleukin-8 response by Chlamydia trachomatis. Cell Microbiol 8:1768–1779. doi:10.1111/j.1462-5822.2006.00747.x 16803583

[48] Cheng W, Shivshankar P, Zhong Y, Chen D, Li Z, Zhong G. 2008. Intracellular interleukin-1alpha mediates interleukin-8 production induced by Chlamydia trachomatis infection via a mechanism independent of type I interleukin-1 receptor. Infect Immun 76:942–951. doi:10.1128/IAI.01313-07 18086816 PMC2258806

[49] Kimmey JM, Campbell JA, Weiss LA, Monte KJ, Lenschow DJ, Stallings CL. 2017. The impact of ISGylation during Mycobacterium tuberculosis infection in mice. Microbes Infect 19:249–258. doi:10.1016/j.micinf.2016.12.006 28087453 PMC5403610

[50] Ritchie KJ, Hahn CS, Kim KI, Yan M, Rosario D, Li L, de la Torre JC, Zhang D-E. 2004. Role of ISG15 protease UBP43 (USP18) in innate immunity to viral infection. Nat Med 10:1374–1378. doi:10.1038/nm1133 15531891

[51] Misaghi S, Balsara ZR, Catic A, Spooner E, Ploegh HL, Starnbach MN. 2006. Chlamydia trachomatis-derived deubiquitinating enzymes in mammalian cells during infection. Mol Microbiol 61:142–150. doi:10.1111/j.1365-2958.2006.05199.x 16824101

[52] Pruneda JN, Bastidas RJ, Bertsoulaki E, Swatek KN, Santhanam B, Clague MJ, Valdivia RH, Urbé S, Komander D. 2018. A Chlamydia effector combining deubiquitination and acetylation activities induces Golgi fragmentation. Nat Microbiol 3:1377–1384. doi:10.1038/s41564-018-0271-y 30397340 PMC6319605

