# Supplementary figures and images for: Chlamydia-driven ISG15 expression dampens the immune response of epithelial cells independently of ISGylation
Source: mBio. 2024 Sep 30;15(11):e02401-24. doi: 10.1128/mbio.02401-24 (PMC11559041; doi:10.1128/mbio.02401-24)

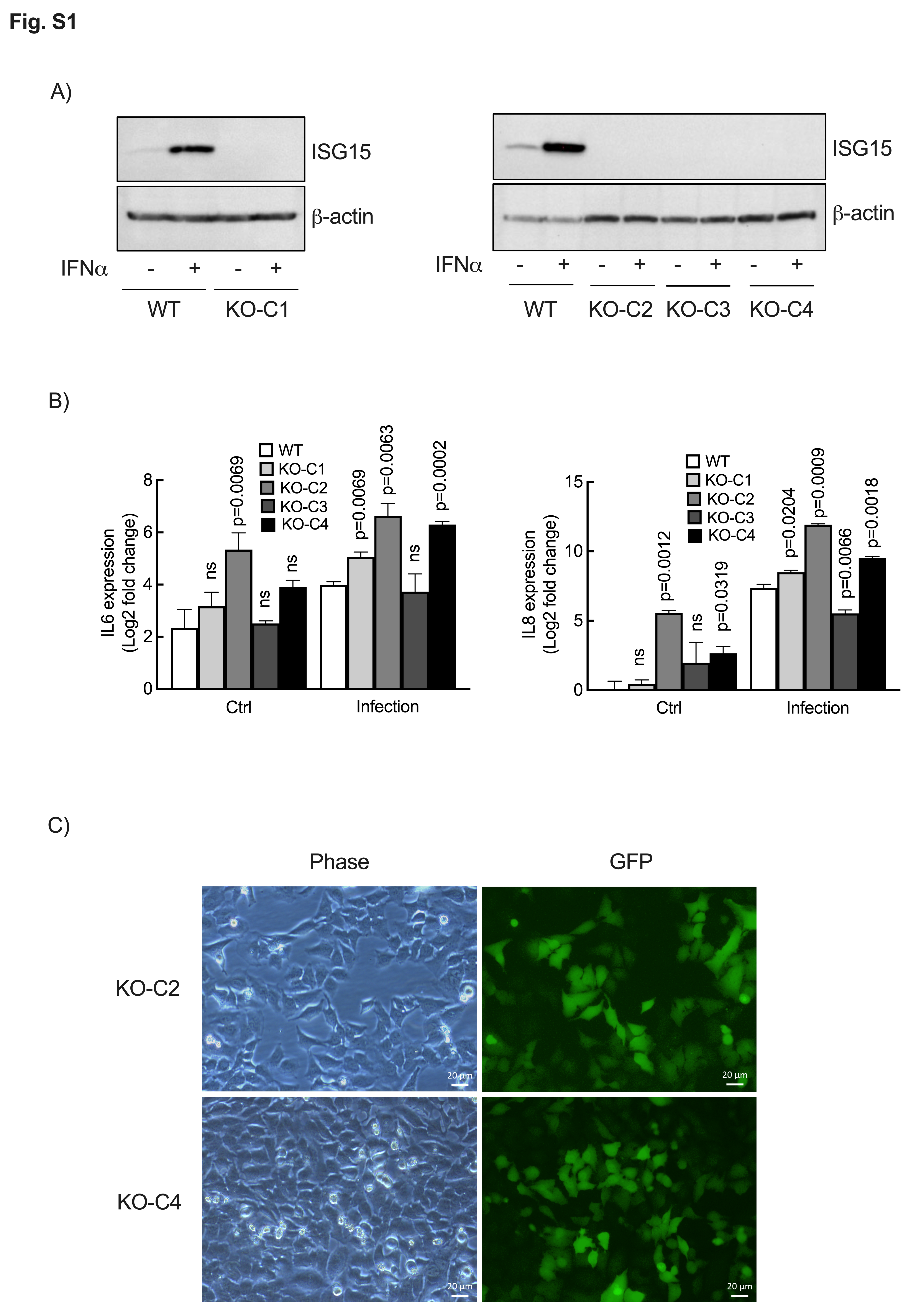

Supplement: Figure S1 — Characterization of ISG15-KO cells. [file mbio.02401-24-s0001.tif]

**Fig. S2 (A)**

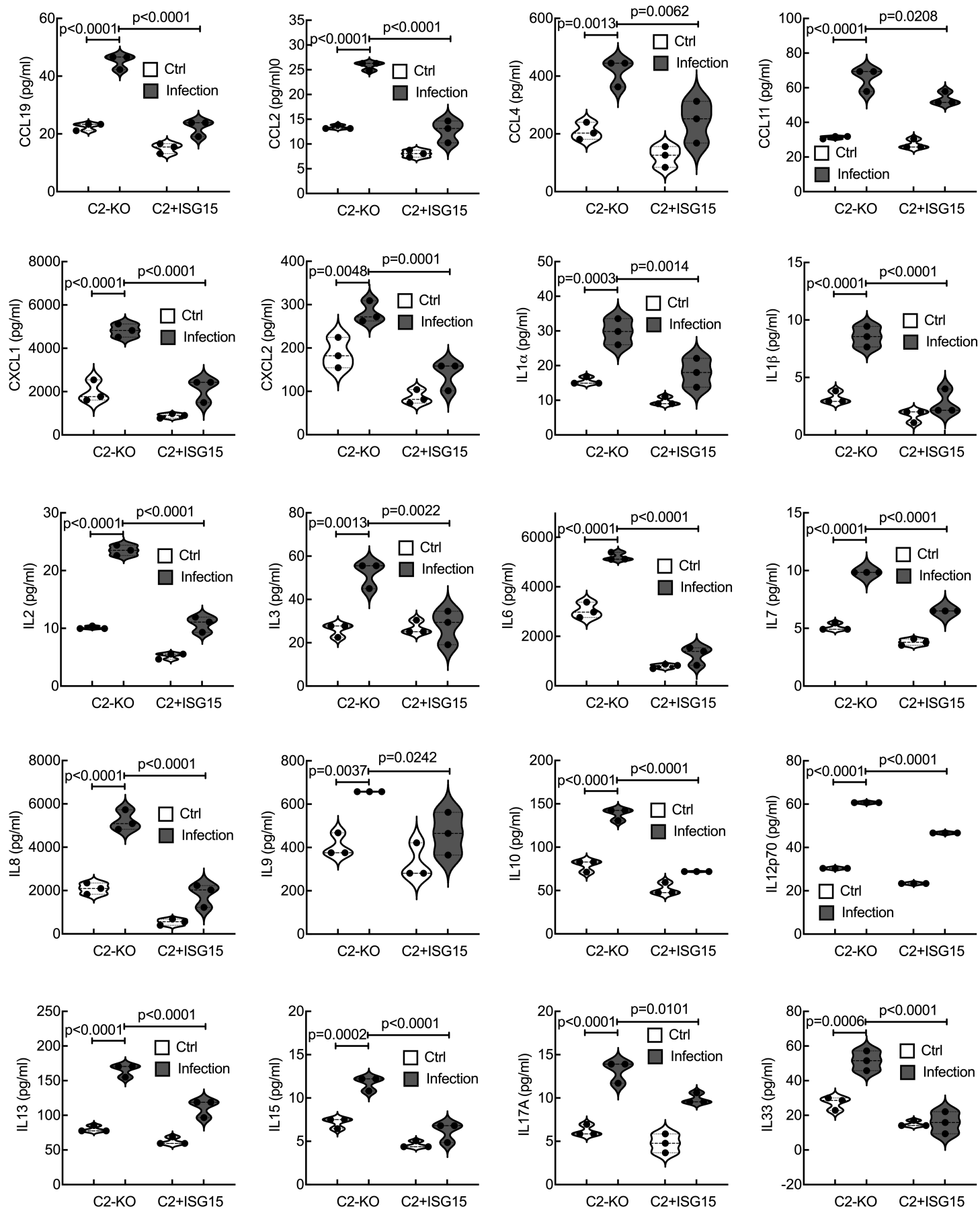

Fig. S2 (A) Continue

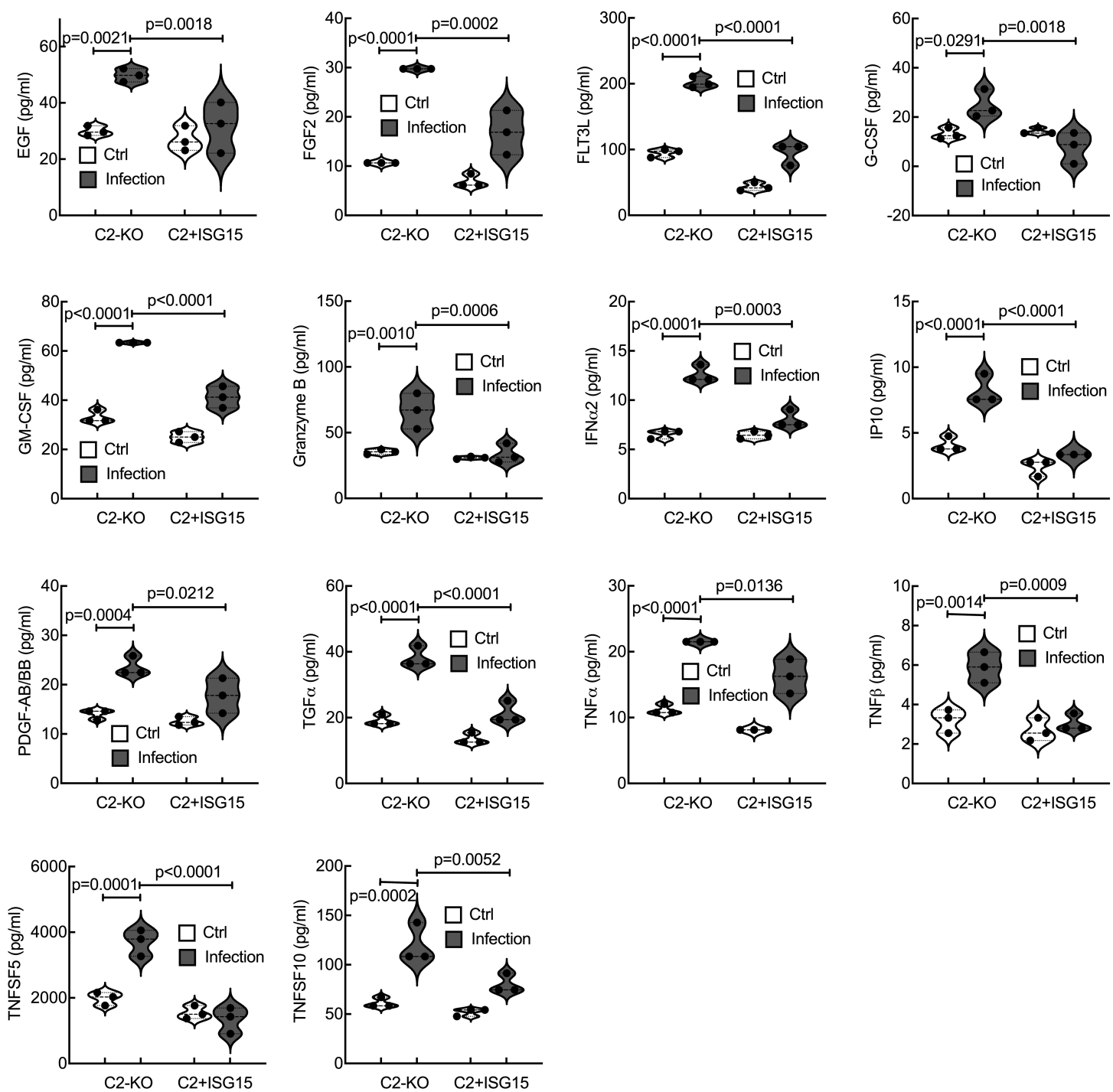

Fig. S2

(B)

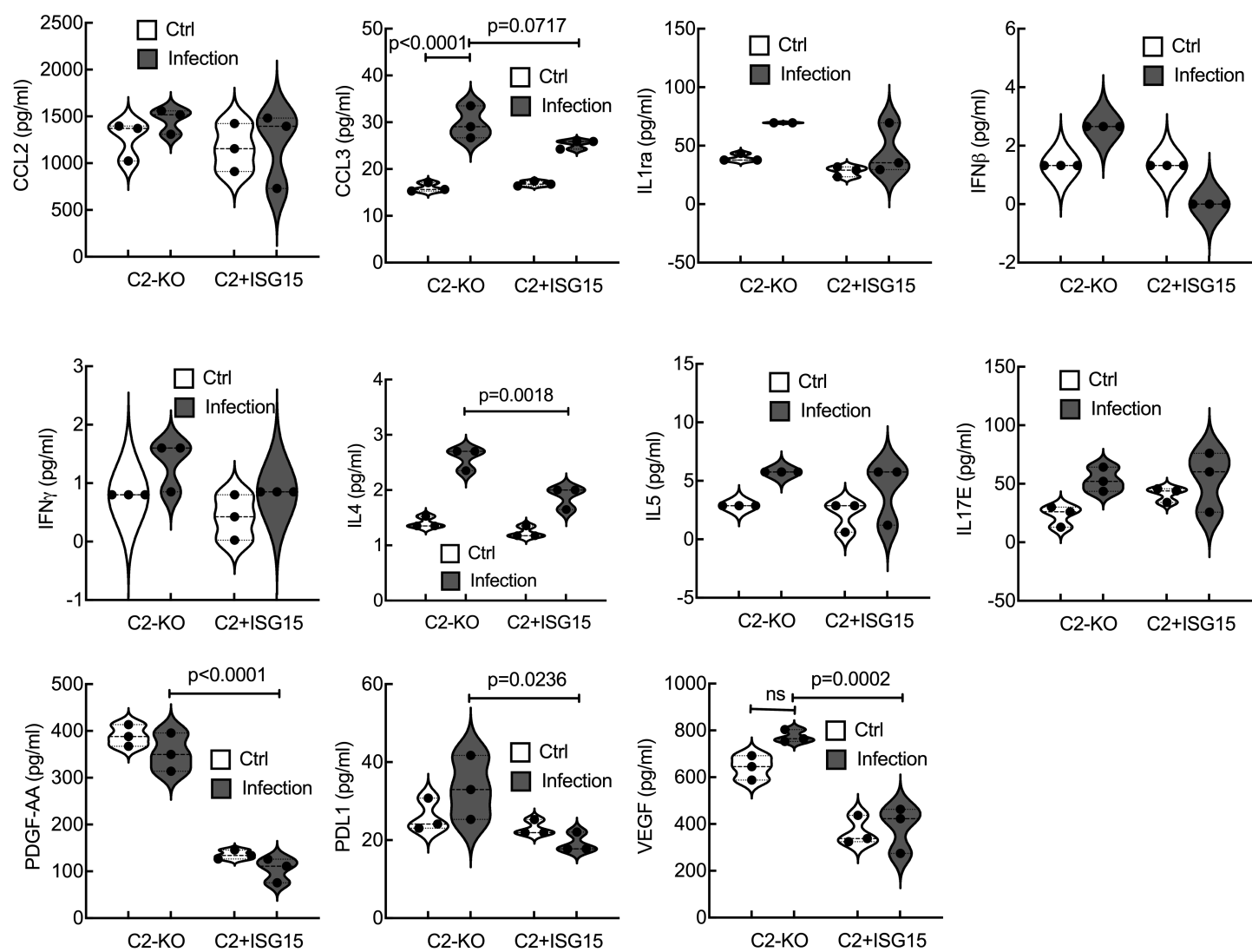

Supplement: Figure S2 — ISG15 dampens the host immune response to C. trachomatis infection. [file mbio.02401-24-s0002.pdf]

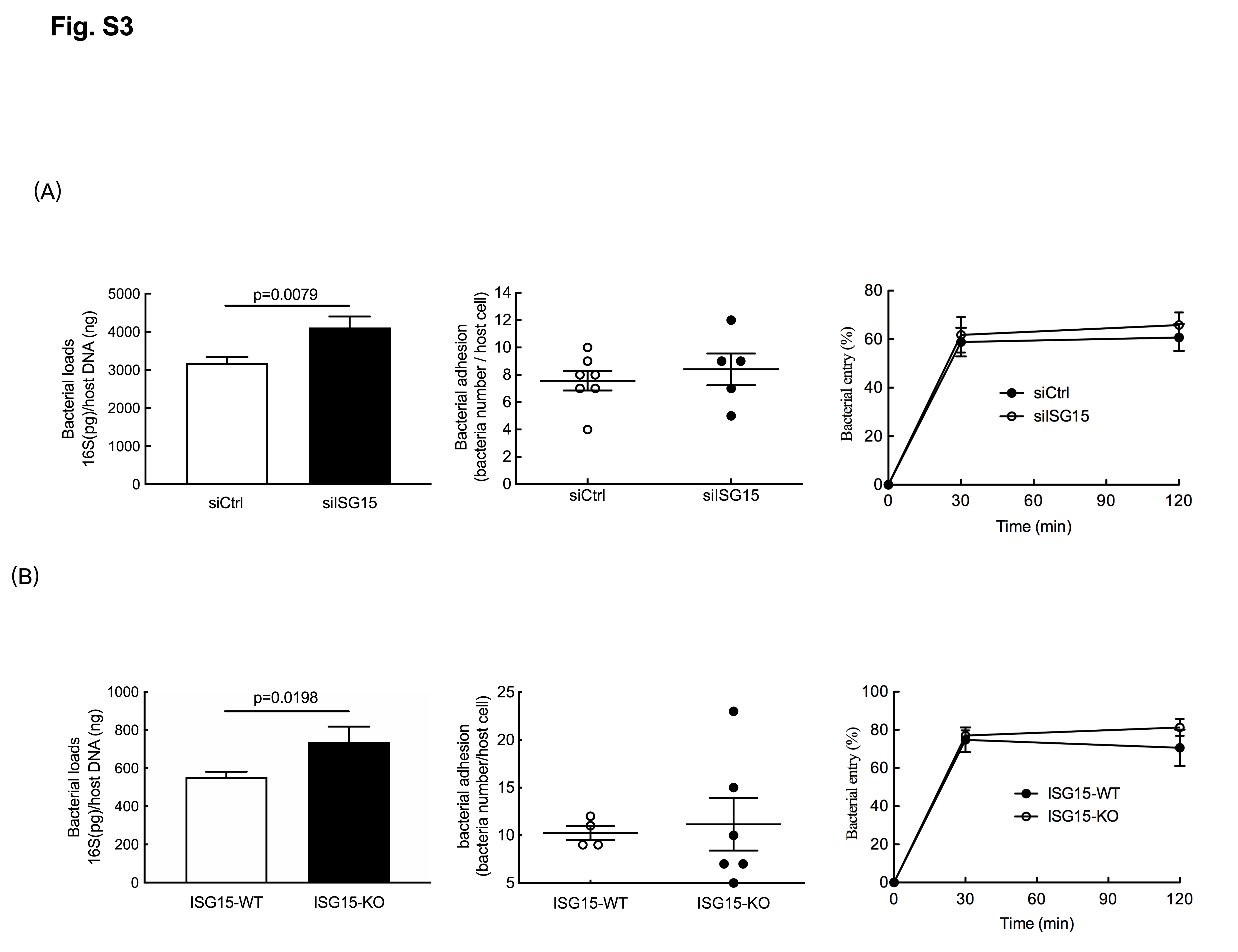

Supplement: Figure S3 — ISG15 depletion favors bacterial growth. [file mbio.02401-24-s0003.tiff]

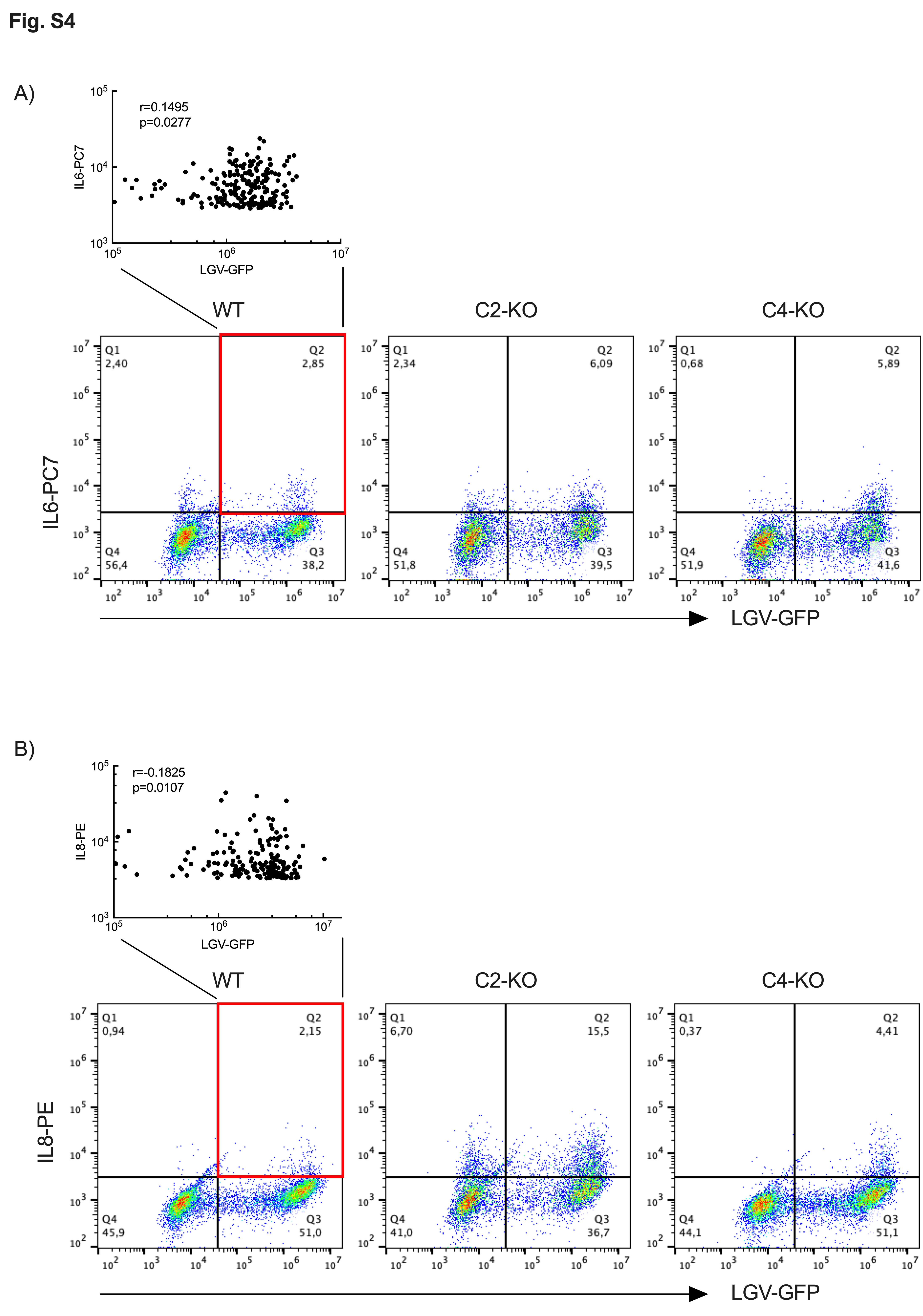

Supplement: Figure S4 — Single-cell analysis of bacterial load and cytokine production. [file mbio.02401-24-s0004.tiff]

Fig. S5

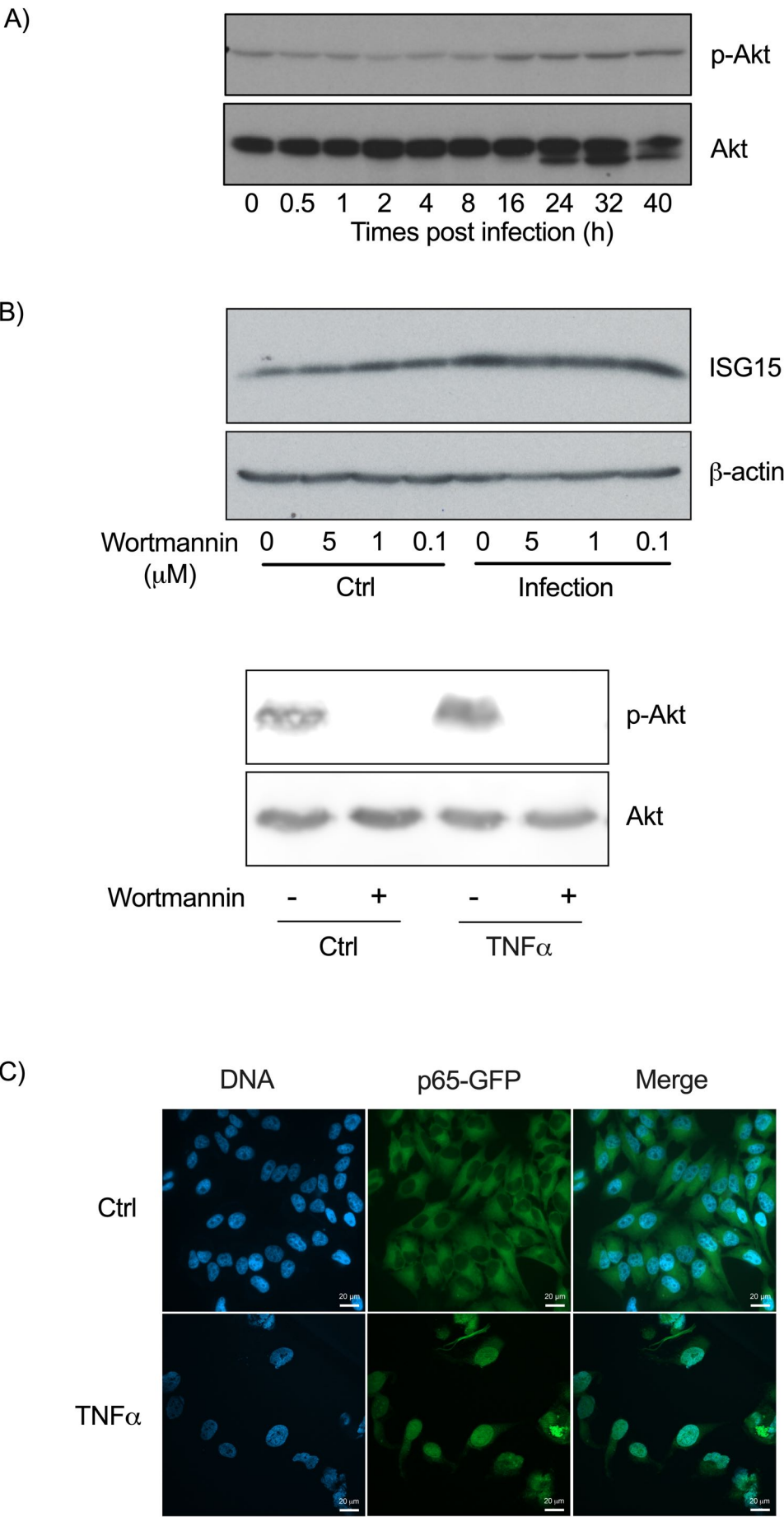

C) continue

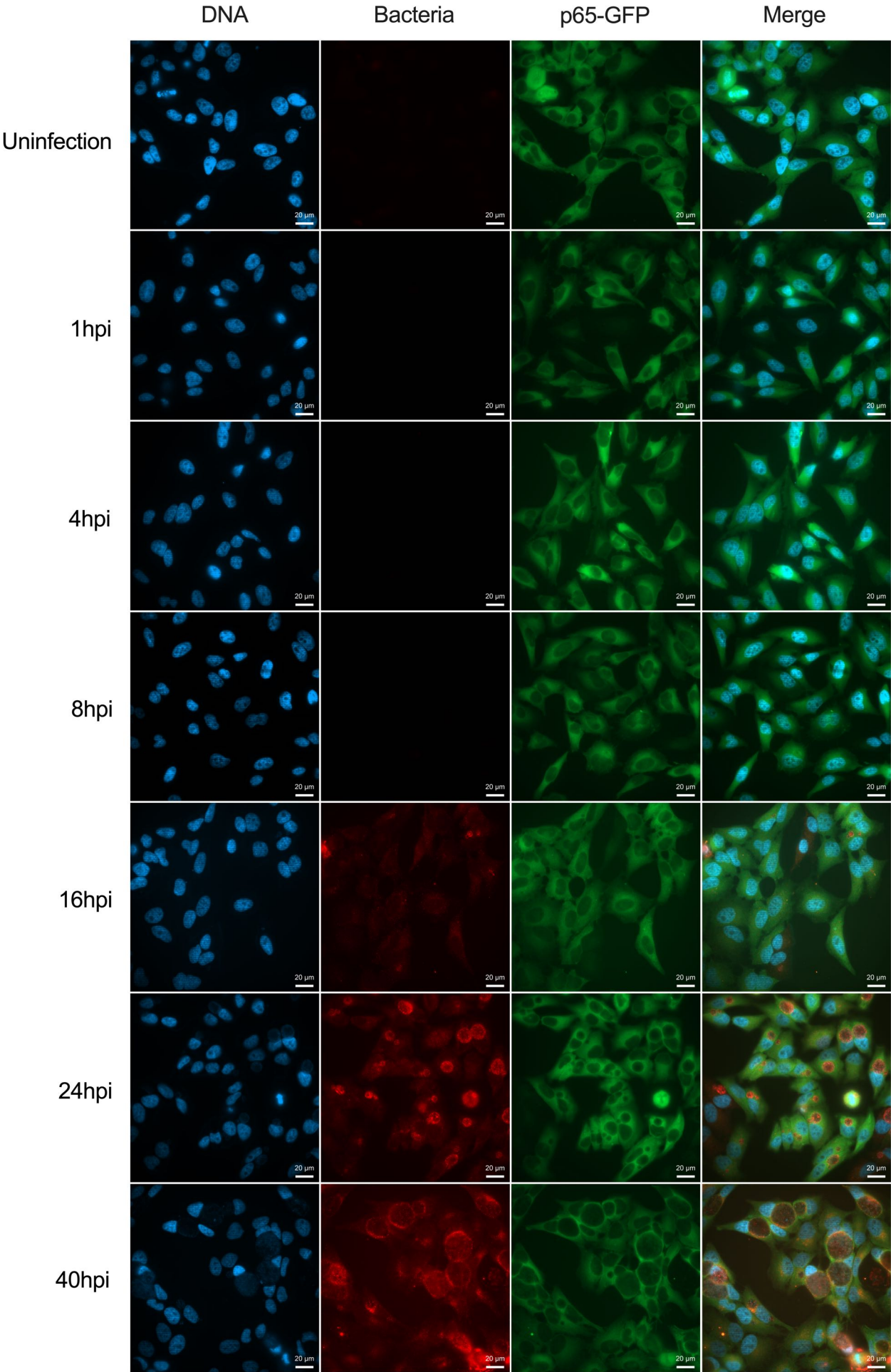

Supplement: Figure S5 — PI3K/Akt and NF-κB signaling pathways are not involved in Chlamydia-induced ISG15 synthesis by epithelial cells. [file mbio.02401-24-s0005.pdf]

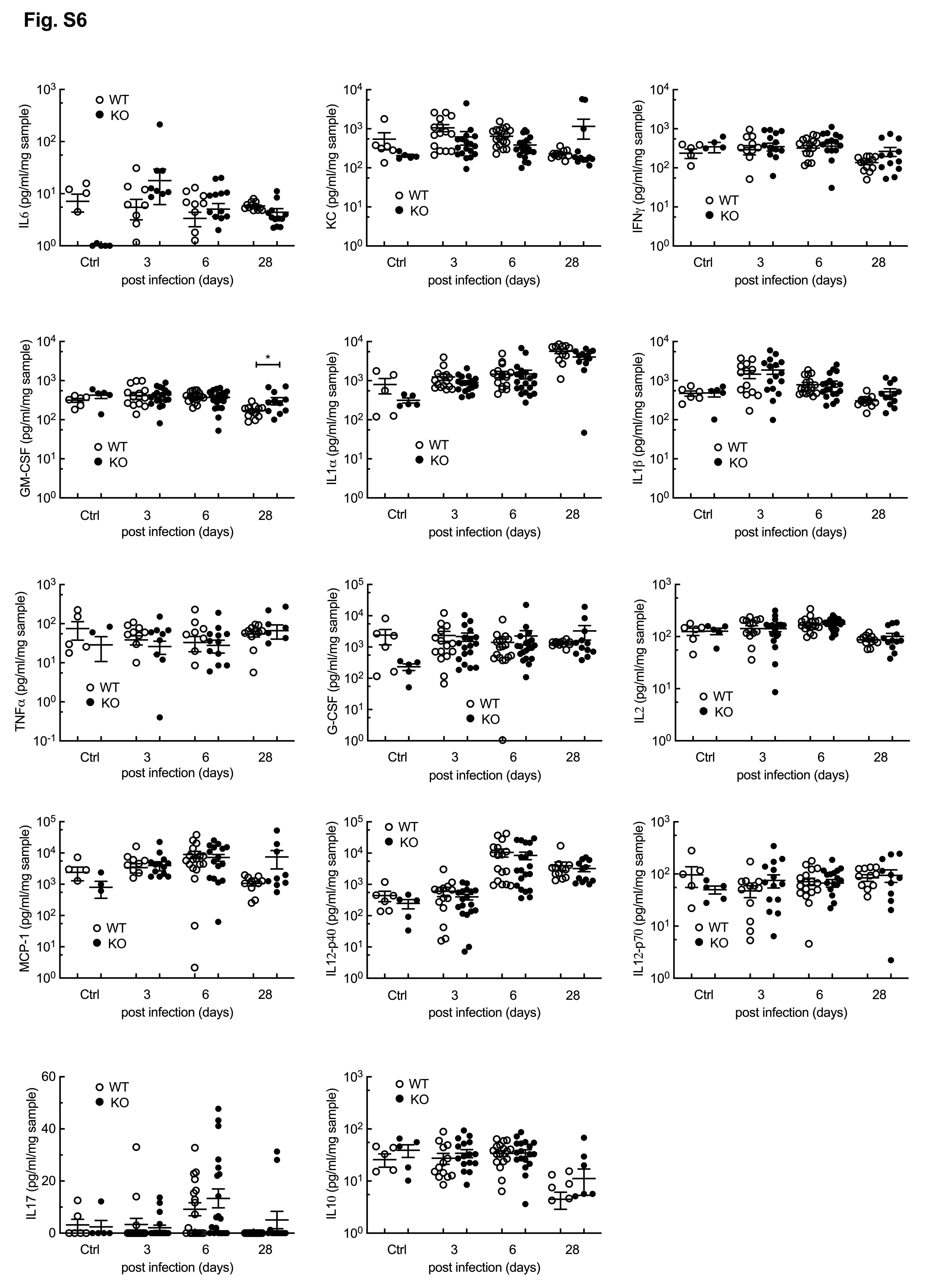

Supplement: Figure S6 — Absence of ISG15 delays C. trachomatis clearance and exacerbates tissue damage. [file mbio.02401-24-s0006.tiff]
