# Supplementary material for: Chlamydia-driven ISG15 expression dampens the immune response of epithelial cells independently of ISGylation
Source: mBio. 2024 Sep 30;15(11):e02401-24. doi: 10.1128/mbio.02401-24 (PMC11559041; doi:10.1128/mbio.02401-24)
Supplement: Legends — for supplemental figures. [file mbio.02401-24-s0007.docx]

***Chlamydia*-driven ISG15 expression dampens the immune response of epithelial cells independently of ISGylation**

Yongzheng Wu^1*^, Chang Liu^1^, Chongfa Tang^1,2,3^, Béatrice Niragire^1^, Yaël Levy-Zauberman^4^, Cindy Adapen^1^, Thomas Vernay^1^, Juliette Hugueny^1^, Véronique Baud^5^, Agathe Subtil^1*^

1 Institut Pasteur, Université Paris Cité, CNRS UMR3691, Cellular biology of microbial infection, F-75015 Paris, France

2 National Vaccine and Serum Institute, Beijing, China

3 Sorbonne Université, Collège doctoral, F-75005 Paris, France

4 Service de Chirurgie gynécologique, Institut Mutualiste Montsouris, F-75014, Paris, France

5 Laboratoire NF-κB, Differentiation and Cancer, Université Paris Cité, F-75006 Paris, France

Short title : ISG15 dampens inflammation in epithelial cells

*corresponding authors: [yongzheng.wu@pasteur.fr](mailto:yongzheng.wu@pasteur.fr); [agathe.subtil@pasteur.fr](mailto:agathe.subtil@pasteur.fr)

**Legends**

Fig. S1 Characterization of ISG15-KO cells. A) ISG15-KO clones and ISG15-WT cells were incubated with IFNα (10 ng/ml) for 24 h or infected with *C. trachomatis* for 42 h before measuring ISG15 levels on whole cell lysates by western blot. B) ISG15-KO and ISG15-WT cells were infected with *C. trachomatis* for 42 h. After infection, the transcripts of pro-inflammatory cytokines *IL6* and *IL8* were examined. Unpair t test was performed to compare the indicated clones with non-infected and infected WT cells, respectively, and the p-values are shown. C) ISG15-KO C2 and C4 cells were complemented for *ISG15* expression. After puromycin selection, cellular pools were imagined in the green channel, as GFP is co-expressed with ISG15. The results are representative of 2 independent experiments.

Fig. S2 ISG15 dampens the host immune response to *C. trachomatis* infection. *ISG15*-KO Hela cells (clone C2) complemented or not with ISG15 was infected with or not with *C. trachomatis* (MOI=1). Classical actors of the immune response were quantified by ELISA in the culture supernatant 42 hpi. Panel (A) displays the extracellular levels of the proteins induced by infection in an ISG15-sensitive manner, panel (B) displays the extracellular levels of the proteins tested that did not follow this pattern. One-way ANOVA was performed for statistical analysis.

Fig. S3 ISG15 depletion favors bacterial growth. A) HeLa cells transfected with siISG15 or irrelevant oligonucleotides were infected with *C. trachomatis* for 42 h followed by the quantification of bacteria loads using qPCR (left panel). For bacterial binding study, pre-chilled siISG15-treated or non-treated HeLa cells were incubated with *Chlamydia* LGV-L2^IncD^GFP (MOI=30) at 4 °C for 4 h. After washing, cells were fixed, stained nuclear DNA and attached GFP-bacteria were quantified under Deltavision™ microscope (middle panel). For bacterial entry study, siRNA-treated cells seeded on coverslip were infected for 45 min at 4°C. After washes, the cells were incubated at 37 °C for the indicated times before staining extracellular bacteria and bacterial entry was analyzed as described in the methodology section (right panel). B) The bacterial binding and entry studies were performed as that in (A) in ISG15-deficient and WT MEFs. Three independent experiments for the studies of bacterial load were performed. The binding and entry studies are representative of two independent experiments. Each dot in the right panels represents quantification of one field. Unpaired t-test was conducted to compare bacterial burdens.

Fig. S4 Single cell analysis of bacterial load and cytokine production. HeLa WT cells (left panels) or *ISG15*-KO clone C2 & C4 cells (middle and right panels) were infected or not with LGV-L2^IncD^GFP bacteria (0.1 μl) for 30 h (in the presence of brefeldin A for the last 6 h) before fixation and staining with PC7-conjugated anti-IL6 (A) or PE-conjugated anti-IL8 (B) antibodies. The LGV^+^/IL6^+^ or LGV^+^/IL8^+^ population (red rectangles) in HeLa WT cells was further analyzed to compare bacterial load and cytokine level. Pearson’s correlation coefficient (r) and P-value were calculated with Prism 9 with a two-tailed P-value.

Fig. S5 PI3K/Akt and NF-κB signaling pathways are not involved in *Chlamydia*-induced ISG15 synthesis by epithelial cells. A) HeLa cells were incubated with *C. trachomatis* (MOI = 1). Akt phosphorylation was examined by western blot at the indicated times post infection. B) HeLa cells were pre-treated with wortmannin (at indicated concentration in upper panel or at 5 μM in lower panel) for 30 min prior to TNFα treatment (10 ng/ml for 1 h) or *Chlamydia* infection (MOI = 1 for 40 h). ISG15 expression (upper panel) was determined by western blot 40 hpi, and Akt phosphorylation (lower panel) was checked 1 h post TNFα stimulation. C) HeLa cells constitutively expressing p65-GFP fusion protein under the control of the EF-1α promoter were incubated with TNFα (10 ng/ml) for 15 min or with *Chlamydia* (MOI=1) for the indicated time, prior to fixation and immunofluorescence analysis to monitor the nuclear translocation of p65. The data represent two independent experiments.

Fig. S6 Absence of ISG15 delays *C. trachomatis* clearance and exacerbates tissue damage. ISG15-KO and wild-type mice were infected with *C. trachomatis* by introducing bacteria into the uterine horn. At the indicated times post infection, animals were sacrificed to harvest the upper FGT. The pro-inflammatory cytokines/chemokines in the upper FGT were determined by multi-plex ELISA. Each dot represents one animal. Unpaired t-tests between animal groups at each time point were performed.
