# Supplementary material for: Chlamydia-driven ISG15 expression dampens the immune response of epithelial cells independently of ISGylation
Source: mBio. 2024 Sep 30;15(11):e02401-24. doi: 10.1128/mbio.02401-24 (PMC11559041; doi:10.1128/mbio.02401-24)
Supplement: Table S1 — Primers. [file mbio.02401-24-s0008.docx]

Table S1. The sequence of primers used for real-time quantitative PCR

|  | sense | anti-sense |
| --- | --- | --- |
| hactin | GGACTTCGAGCAAGAGATGG | GCAGTGATCTCCTTCTGCATC |
| hIL6 | CACACAGACAGCCACTCACC | CATCCATCTTTTTCAGCCATC |
| hIL8 | AGCCTTCCTGATTTCTGC | GCCCTCTTCAAAAACTTCTC |
| hISG15 | CGCAGATCACCCAGAAGATCG | TTCGTCGCATTTGTCCACCA |
| hcGas | GGGAGCCCTGCTGTAACACTTCTTAT | CCTTTGCATGCTTGGGTACAAGGT |
| *Chlamydia* 16S | TGGATGAGGCATGCAAGTC | TACTAACCCTTCCGCCACTAAA |
| mactin | AGCTGTGCTATGTTGCTCTAGACTT | CACTTCATGATGGAATTGAATGTAG |
| mIL6 | TAGTCCTTCCTACCCCAATTTCC | TTGGTCCTTAGCCACTCCTTC |
| mKC | ﻿﻿﻿AACGCTGGCTTCTGACAAC | ﻿﻿﻿TTCGCACAACACCCTTCTAC |
